# Comparative Analysis of Species-Specific Hepatocyte Function and Drug Effects in a Liver Microphysiological System PhysioMimix LC12 and 96-Well Plates

**DOI:** 10.1021/acsptsci.5c00554

**Published:** 2025-10-03

**Authors:** Chander K. Negi, Courtney Sakolish, Han-Hsuan D. Tsai, Katharina Nitsche, Han Gang, Piyush Bajaj, Stephen S. Ferguson, Jason P. Stanko, Philip Hewitt, David A. Kukla, Sarah M. Lloyd, Remi Villenave, Ivan Rusyn

**Affiliations:** † Department of Veterinary Physiology and Pharmacology, 2655 Texas A&M University, College Station, Texas 77843, United States; ‡ Division of Toxicology, 4508 Wageningen University and Research, Wageningen, Gelderland 6708 WE, The Netherlands; § Department of Epidemiology and Biostatistics, 2655 Texas A&M University, College Station, Texas 77843, United States; ∥ Global Investigative Toxicology, Preclinical Safety, 2194 Sanofi, Cambridge, Massachusetts 02141, United States; ⊥ Division of Translational Toxicology, National Institute of Environmental Health Sciences, Research Triangle Park, North Carolina 27709, United States; # Chemical and Preclinical Safety, 2792 Merck KGaA, Darmstadt 64293, Germany; ∇ Development Sciences, 359181 AbbVie Inc., North Chicago, Illinois 60064, United States; ○ Roche Innovation Center Basel, F. Hoffmann-La Roche Ltd, Roche Pharma Research and Early Development, 4070 Basel, Switzerland

**Keywords:** cross-species DILI, PhysioMimix LC12, microphysiological system

## Abstract

Drug-induced liver injury (DILI) remains a challenge in drug development, and interspecies differences in liver toxicity represent a need where comparative analyses may inform preclinical safety study design. In vitro testing for species-specific liver effects, especially in complex models such as microphysiological systems (MPS), may help predict toxicity before advancing from animal to human studies, or derisk spurious findings in preclinical species. This study assessed the utility of the perfusion-based PhysioMimix LC12 MPS as compared to 2D cultures and evaluated species-specific DILI using primary hepatocytes from human, monkey, rat, and dog. Functional, phenotypic, and transcriptional profiles were evaluated for up to 14 days. Also, cells were exposed to species-specific hepatotoxicants such as bosentan (BOS), fialuridine (FIAU), and a common hepatotoxicant for all species, chlorpromazine (CPZ)—in both PhysioMimix LC12 and traditional 2D cultures. Hepatocytes in PhysioMimix LC12 showed more stable albumin and urea production as compared to 2D cultures. Concentration–response studies with CPZ, BOS, and FIAU were performed in 2D; then, repeated (5 × every 2 days) exposures to sub-100 × *C*_max_ concentrations were tested in PhysioMimix LC12. Species-specific differences in cellular and molecular effects of the drugs were observed in both models; data from PhysioMimix LC12 were reflective of the expected effects in both animals and humans. Still, variability and low throughput are limitations of MPS for prospective studies of species-specific responses. Overall, this study confirms the utility of liver safety studies using PhysioMimix LC12 and also provides suggestions for experimental designs to overcome the limitations of more complex test systems.

## Graphical abstract

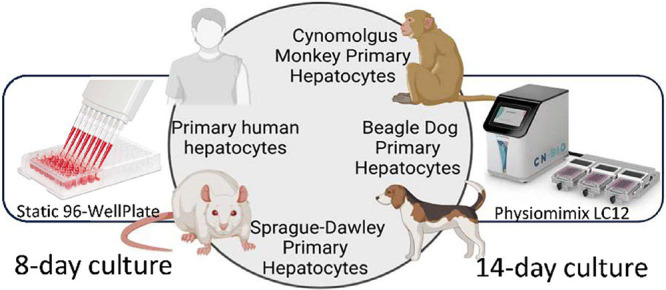

1

The regulatory agencies worldwide, including the US Food and Drug Administration (US FDA) and the European Medicines Agency (EMA), mandate rigorous testing in both preclinical and clinical phases of drug development^1^ and monitoring in postmarketing.^2^ Studies in animals comprise the large part of these regulatory requirements—they are designed to identify potential toxicities, pharmacokinetic profiles, as well as establish the therapeutic index and safety margins to inform the first-in-human dosing for clinical trials.^1^ Despite the critical role that animal testing plays in drug development, they suffer from notable shortcomings, including a limited translatability to human biology that may preclude their use as preclinical testing tools.^3^ For example, drug-induced liver injury (DILI) is a substantial challenge in both drug development and clinical practice.^4,5^ DILI is a frequent cause for attrition of promising new drug candidates and postmarketing withdrawal of prescription drugs.^6^ Species-specific differences in liver physiology, metabolism, and immune responses can result in mispredictions of human safety.^7^

In some cases, drugs that were found to have DILI risk in humans showed no evidence of toxic responses in preclinical animal studies, exemplifying the concern for the potential lack of concordance between preclinical and clinical findings.^7,8^ The majority of human DILI cases were observed either late in development or not until the drug was approved, leading to costly late-stage attrition or market withdrawal.^9^ For example, fialuridine (FIAU), an investigational nucleoside analogue, exhibited no liver toxicity in preclinical studies but led to fatal hepatotoxicity in five subjects during phase II clinical trial;^10,11^ its potential liver toxicity is thought to involve depletion of mitochondrial DNA, steatosis, and cholestasis.^12,13^ Bosentan (BOS) is an antagonist of endothelin receptor that causes liver injury and cholestasis in humans via bile salt export pump inhibition;^14,15^ toxic effects have not been found in the rat, despite transporter inhibition, and variable effects were observed at 10 × *C*_max_ in a dog study.^16^ Additionally, chlorpromazine (CPZ) is a widely used neuroleptic drug for the treatment of schizophrenia; it has been shown to be hepatotoxic in both humans and rats by a mixed cytotoxic and cholestatic mechanism, albeit at very high doses.^17,18^ CPZ causes hepatotoxicity in rodents when combined with pro-inflammatory stimuli.^19^ These examples highlight the limitations with translating liver findings from preclinical species to humans because of the potential differences in drug metabolism, immune system response to mild liver injury, and liver physiology.^20^ These gaps in predictive accuracy of preclinical cell- and animal-based models underscore the need for improved methods that may better mimic human liver function as well as enable improved translational studies during preclinical development.^21–23^

Numerous advancements in modeling the human liver in vitro have been achieved in the past decade.^24–26^ Most recent examples include hepatic spheroids and complex in vitro culture platforms, such as microphysiological systems (MPSs). These models show great promise in better replicating human liver responses to drugs and improving the prediction of clinical DILI.^27,28^ Specifically, MPSs have been developed to mimic the structural, functional, and the complex biological microenvironments of the liver, such as media flow to facilitate tissue oxygenation and hepatocyte zonality.^29–31^ Multiorgan MPS can also mimic physiological interactions between liver and other tissues and enable in vitro studies of drug distribution and metabolism, as well as the toxicity caused by drug metabolites.^32,33^ Overall, MPS have emerged as a potentially transformative tool for drug development, safety evaluation, and mechanistic studies of complex biological processes using human cells as an alternative to studies in animals.^34–36^ Also, these platforms have the potential to accelerate studies of interindividual variability and testing new drug modalities.^31^

Many liver MPSs have been developed in the past decade; several of these are available commercially.^30,37,38^ These models typically incorporate primary human hepatocytes, induced pluripotent stem cell (iPSC)-derived hepatocytes, or liver cell lines such as HepaRG, with or without other liver cells.^28,37–41^ Liver MPSs have also been used to demonstrate interspecies differences in responses to drugs using rat, dog, and human liver cells.^39^ Indeed, the ability of liver MPS to recapitulate human-specific responses holds promise for regulatory evaluations in drug safety testing, refining, and potentially reducing the need for preclinical animal studies as well as improving translational relevance.^28,42^

Still, few studies have evaluated the robustness and reproducibility of liver MPS in cross-species study designs or compared them side by side to more traditional in vitro liver models to increase confidence for wider adoption and regulatory qualification.^28,40,43,44^ Standardized protocols, interlaboratory comparisons, and alignment with regulatory expectations are needed to establish confidence in their predictive capability and facilitate use in drug safety assessments.^45,46^ Accordingly, this study aimed to characterize the phenotypic, functional, and transcriptional biomarkers of both basal and drug-treated cultures of species-specific primary hepatocytes from human, monkey, rat, and dog. These cells were cultured in a standard 2D format or in the perfusable PhysioMimix LC12 MPS (Figure 1). This MPS was selected based on its relatively higher throughput (12 conditions on one plate with up to 6 plates that can be ran using one controller unit) among other available liver MPS. In addition, this model has been tested for its robustness and reproducibility in several previous studies and was shown to be a promising model.^40,47^ We evaluated the robustness of this MPS platform under long-term (up to 2 weeks) culture and also examined species-specific DILI outcomes with known hepatotoxic drugs, including FIAU, BOS, and CPZ.

**Figure 1:**
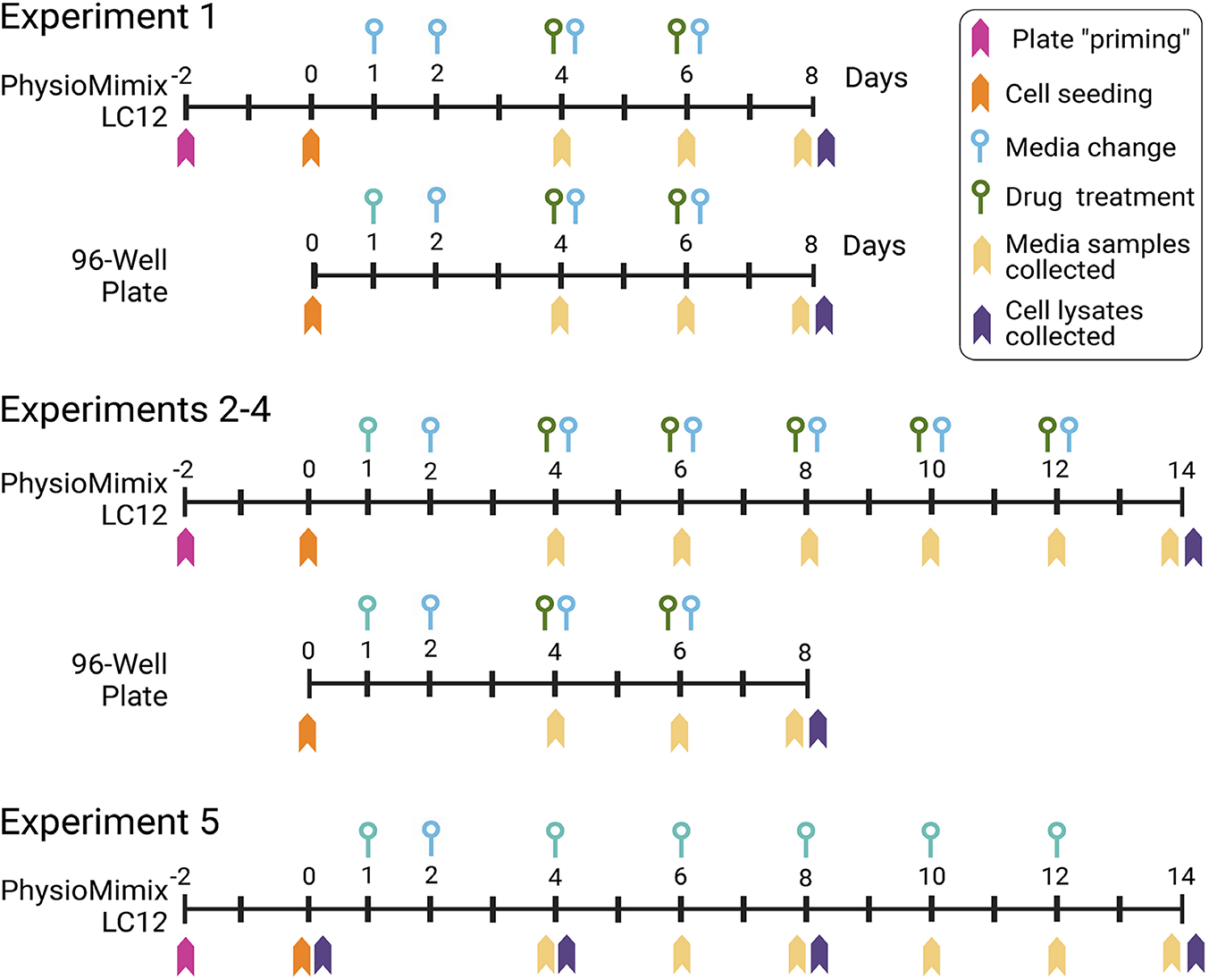
Experimental design for comparative hepatotoxicity assessment in Liver-on-Chip and 2D cultures using human and animal hepatocytes. Primary hepatocytes from human, cynomolgus monkey, rat, and dog were cultured in both MP (PhysioMimix LC12) and conventional 2D monolayer format. The day of hepatocyte seeding was designated as day 0 for all experiments. Drug treatment with chlorpromazine (CPZ), bosentan (BOS), and fialuridine (FIAU) was initiated on day 4 of culture, with media containing fresh drug replaced every other day. Samples were collected on different days as indicated for various end points.

## 2 Results and Discussion

### 2.1 Characterization of Species-Specific Hepatocyte Function in 2D and PhysioMimix LC12 MPS

The key element of the PhysioMimix LC12 liver MPS is the microchanneled (∼300) scaffold that is designed to facilitate the formation of perfusable spheroidal aggregates of hepatocytes and enable constant recirculation of the oxygenated cell culture medium.^47–49^ Tissues that are formed in these microchannels can be assessed using optical microscopy to verify seeding quality.^40^ Representative PhysioMimix LC12 scaffold images and quantification of the microchannel coverage by the cells over time (4, 8, and 14 days) are shown in Figure 2.

**Figure 2:**
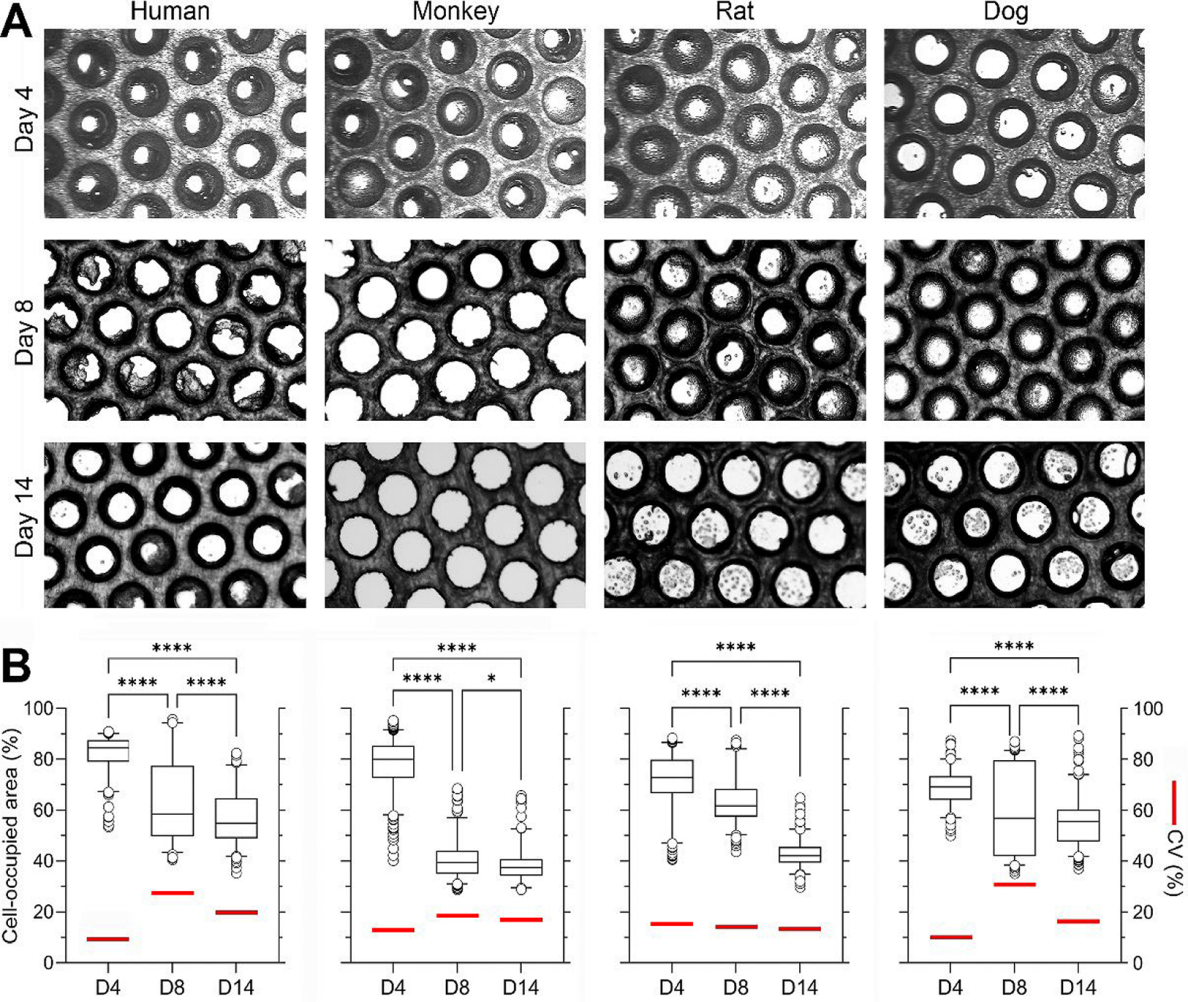
Phenotypic characterization of species-specific hepatocytes density in PhsyioMimix LC12 MPS. (A) Representative bright-field images of hepatocytes in the scaffold in PhysioMimix LC12 from days 4, 8, and 14. (B) Quantification (left *y*-axis) of the microchannel coverage area by the cells over time in the subregions of the representative scaffolds on days 4, 8, and 14 in culture, right *y*-axis shows the coefficient of variability for each microchannel-to-microchannel variability in cell coverage area (thick red horizontal line). Box-and-whisker plots show the median (line), interquartile range (box), and range (whiskers). Asterisks (*) denote statistical differences between subregions (one-way ANOVA with Dunnett’s multiple comparisons). Significance is indicated as follows: *p < 0.05; ****p < 0.0001.

We tested hepatocytes from 4 species—human, monkey, rat, and dog (Table 1)—and found that on day 4, for all species tested, the hepatocytes were uniformly distributed both along the walls of the scaffold channels and on the surface of the scaffolds. The cell coverage (fraction of cell-covered area to the total area of each microchannel) ranged from 82 ± 8% for human hepatocytes to 69 ± 7% for dog hepatocytes. Over time, a significant decrease in the coverage was observed on day 14, and it was the highest (57 ± 11%) in human hepatocytes and the lowest (38 ± 7%) in monkey hepatocytes. The microchannel-to-microchannel variability in cell coverage was 10–15% on day 4 and generally increased with time, except for rat hepatocytes. While previous studies also used scaffold images to explain poor performance of some microtissues,^47^ to our knowledge, the time course in cell coverage in this MPS has not been previously reported. One previous study estimated that cell seeding efficiency in this model on day 1 varies from 50 to 60%;^49^ thus, our data may be used to conclude that by day 14, an even smaller fraction of seeded hepatocytes remains inside scaffolds. The observed time-dependent cell loss that also varies among species could be attributed to incomplete seeding efficiency, likely resulting from a combination of inadequate hepatocyte attachment to the channel surface and mechanical stress during medium recirculation.^49^ Additionally, hepatocytes are highly sensitive to their microenvironment, and prolonged culture can lead to the loss of critical cell–matrix and cell–cell interactions that are required for maintaining long-term function and matrix attachment. It should be noted that for the future use of this model for cross-species comparisons, not only should the loss of cells be considered as a limitation but also the variability in coverage in the scaffolds may result in the cells experiencing different levels of shear stress.

**Table 1:** Hepatocytes Used in This Study

cell type	catalog and lot number	donor sex	cell vendor	post-thaw cell viability (%)
primary human hepatocytes	HMCPIS	female	Thermo Fisher	83.8–87.4
	lots # HU8373			
cynomolgus monkey primary hepatocytes	M003055-P	male	BioIVT	85.5–90.7
	lots # JCW			
Sprague–Dawley rat primary hepatocytes	M00005-P	male	BioIVT	73.9–85.8
	lots # FIM			
Beagle dog primary hepatocytes	M002055-P	male	BioIVT	76.2–86.9
	lots # BTQ			

To determine the functionality of the hepatocytes cultured in MPS, we measured specific liver function markers such as secreted albumin and urea for up to 14 days, and the values were normalized to the initial seeding density (Figure 3). The data were compared to the same donor-derived species-specific hepatocytes cultured for up to 8 days in 2D on static 96-well plates. For albumin, human, and monkey, hepatocytes in the PhysioMimix LC12 showed about 3-fold higher levels as compared to the 2D condition on day 4. Levels of albumin in rat and dog hepatocytes on day 4 were comparable between the 2D and MPS models (Figure S1). In all species, albumin levels gradually declined with time. In human and monkey hepatocytes, albumin decreased to about one-third of the peak level by day 8 and remained at that level up to 14 days in PhysioMimix LC12; these steady-state levels were greater than in 2D cultures after 8 days. Rat hepatocytes produced similar levels of albumin over 1 week in both models; the levels began to decline after 10 days in the PhysioMimix LC12, albeit no comparison could be made to 2D. Levels of albumin in dog hepatocytes were among the lowest of all species tested even at day 4; they declined rapidly in both culture modalities. Experiment-to-experiment variability in these measurements is shown in Figure S2—most experiments and species were below 30% variability, which was suggested as an acceptable threshold for in vitro liver studies.^31^ However, overall variability in the PhysioMimix LC12 model was generally greater than 2D, especially in experiments with rat hepatocytes, where it ranged from 40 to 80%. Trends in urea production generally followed those for albumin; however, urea levels were comparable among species, with only monkey hepatocytes producing about twice as much urea as cells from other species. The variability in urea measurements was below 30% for all species and for both models.

**Figure 3:**
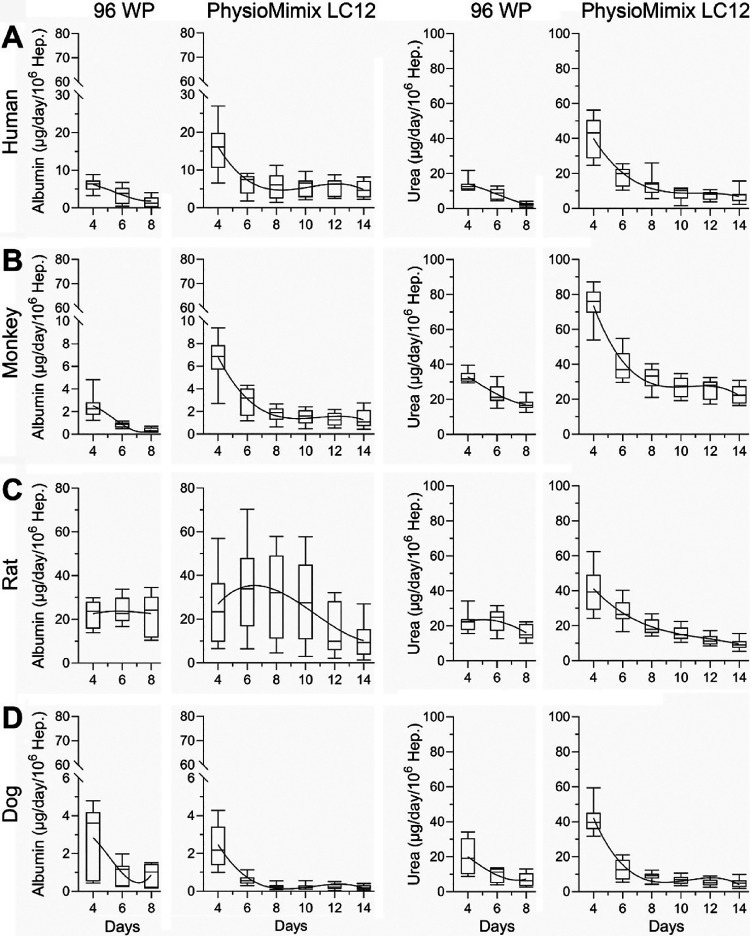
Comparative analysis of the basal levels of albumin and urea secretion in species-specific MPS and 2D culture. Time course of albumin and urea secretion over an 8 day period in 2D static culture and a 14 day period in PhysioMimix LC12 for (A) human, (B) monkey, (C) rat, and (D) dog hepatocytes. The box plots show the median, interquartile range (IQR), and 10th–90th percentiles, capturing the spread and variability in albumin and urea secretion levels across different experimental setups.

Other cell viability biomarkers (lactate dehydrogenase (LDH), aspartate transaminase (AST), and alanine aminotransferase (ALT)) were also evaluated (Figures S3–S5). Generally, low levels of these biomarkers were detected, with a trend toward a decline over time, a phenomenon that may be related to a decay in the number of viable cells (Figure 2). Variability was also below 30%, except for the level of LDH in rat hepatocytes, which increased with time, exceeding the 30% threshold after 10 days in culture (Figure S3). Variability in ALT and AST was below 30% for all cells and culture conditions over the duration of the experiments (Figures S4 and S5).

This comparative analysis highlights not only the differences in liver-specific function of hepatocytes when cultured in the dynamic microfluidic platform or traditional static 2D plates but also the similarities across species. We previously compared the functionality of primary human hepatocytes from multiple donors in different liver MPSs and in 2D cultures.^40^ We observed a wide range in both urea and albumin production; both markers were generally below those reported for human liver (albumin 37–105 μg/day/million hepatocytes; urea 56–159 μg/day/million hepatocytes).^31^ In the current study, we used cells from the same human donor that we tested previously in other culture modalities.^40,43^ The basal functionality and trends in both the PhysioMimix LC12 and 2D models were nearly identical to those that were published previously, indicating that this MPS model is reproducible and that donor-specific properties are most impactful on the study outcome. For similar comparisons in other species, we note that liver albumin production (μg/day/million hepatocytes) has been reported in the range of 130–750 for the rat^50,51^ and at ∼160 for the dog;^52^ no studies are available for the monkey. Thus, while we show that hepatocytes from different species exhibit similar declining trends in biosynthetic function in the PhysioMimix LC12, we did observe species differences with rat albumin production being the highest, commensurate with in vivo data. We also note that because the normalization is done to the initial seeding density and it is likely that only 10–20% of hepatocytes remain past 1 week in culture (Figure 2), cells do appear to maintain secretion of liver-specific functional markers for up to 14 days, indicating the feasibility of both repeat-dose studies and the use of albumin and urea as biomarkers of potential adverse effects on the synthetic function of the liver in the PhysioMimix LC12 MPS.

The greater variability observed in the MPS is an important consideration because it has implications for future study designs. While 2D cultures can be easily scaled into multiple 96-well plates, the PhysioMimix LC12 model utilizes a 12-well format and up to 6 plates (a total of 72 conditions) can be run on a single controller. Handling of 6 PhysioMimix LC12 plates is more technically challenging than that of standard multiwell plates. Therefore, understanding how many replicates may be needed for each condition is a critical consideration for the use of this MPS. Our previous sample size estimates for observing time-related differences in basic hepatic function in the experiments with the PhysioMimix LC12 MPS seeded with primary human hepatocytes showed that more than 6 replicates are needed (80% power and *p* < 0.05 significance).^40^ In this study, a similar analysis showed that in human hepatocytes, 4–7 replicates are needed to observe differences between days 4 and 14 for albumin and urea (Table S1) and LDH (Table S2). For 96-well experiments, the numbers of replicates needed to reach statistical significance were largely similar to those in PhysioMimix LC12 MPS. For primary hepatocytes from other species, the number of replicates needed ranged from 3 to 11—highest for the albumin in the rat and lowest for urea across three nonhuman species. These results assume repeat measures, i.e., sampling of the media from the same device over time, highlighting that the collection of media from the same devices (paired analyses) significantly reduces variability. This strategy, only possible in PhysioMimix LC12 MPS because of the large circulating media volume, improves efficiency and enhances the ability to detect time-dependent effects. By incorporating paired analyses, temporal changes can be captured with greater precision and a smaller sample size, providing deeper insights into dynamic behavior of the cells in these platforms over time, which can overcome the low throughput challenge of the current system.

### 2.2 Time-Trend Analysis of Species-Specific Gene Expression in PhysioMimix LC12 MPS

Model-omics characterization of MPS is an important requirement for the adoption of new models by the end-users.^53^ Indeed, gene expression, especially of key phase I and II drug metabolizing enzymes and transporters over a 14 day period, has been recommended as a benchmark for characterizing model performance of liver MPS.^31^ Accordingly, we conducted a separate experiment to test gene expression in the same hepatocytes from four species cultured in the PhysioMimix LC12 model for up to 14 days (Figure 4). When all expressed genes that could be aligned among species (*n* = 12,809) were compared (Figure 4A and Tables S4 and S5), the most prominent difference among samples was between day 0 (cells before plating) and other experimental days. This phenomenon was consistent across all of the species. Also, the overall transcriptomes were largely similar across species with high, intermediate, and low expression genes being largely consistent. This observation may reflect the transition from the cryopreserved state to the metabolically active state.

**Figure 4:**
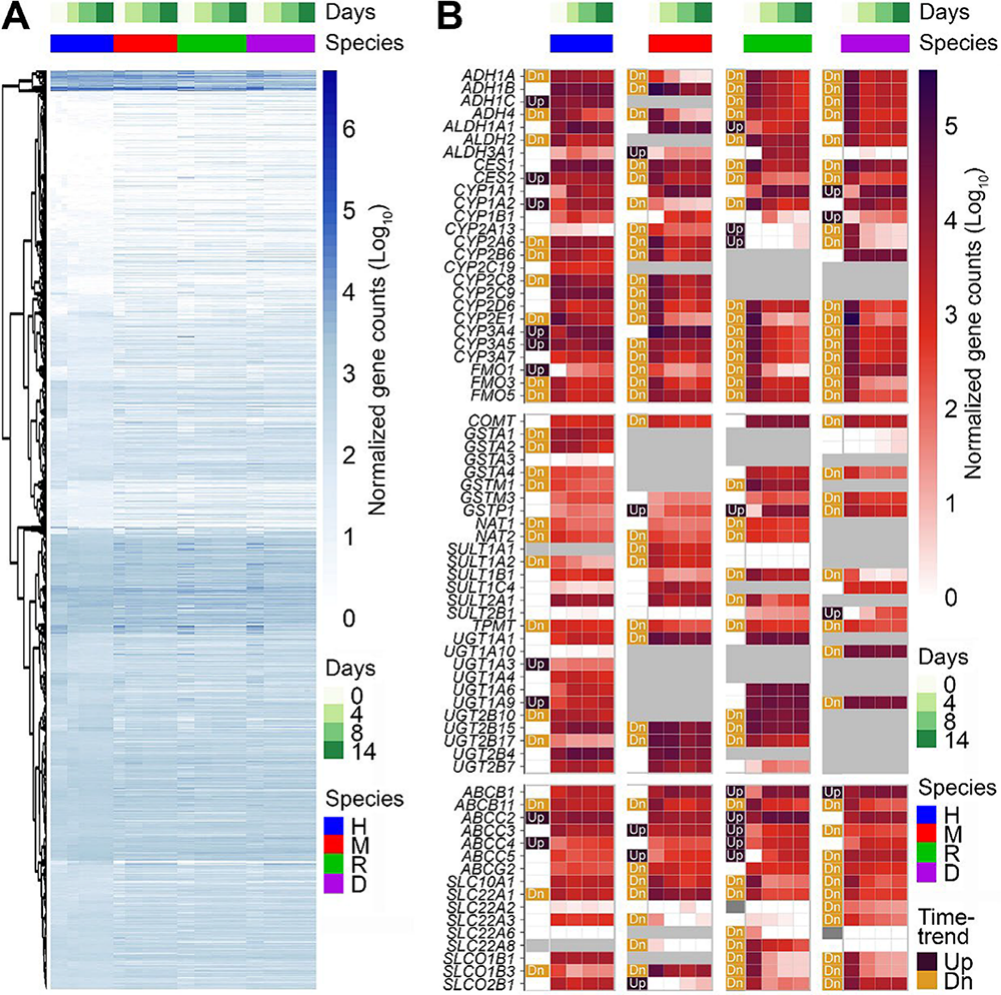
Temporal dynamics of gene expression analysis of species-specific PhysioMimix LC12 MPS. (A) Gene expression analysis of hepatocytes cultured in the PhysioMimix LC12 system comparing temporal profiles across multiple species to assess transcriptomic responses over time. Heatmap depicting normalized counts of all overlapping genes with human orthologs from day 0 (before-plating) to postplating time points (days 4, 8, and 14), highlighting transcriptional changes in response to culture duration. (B) Temporal expression patterns of normalized gene counts were examined for representative ADME-related genes, including cytochrome P450 enzymes (phase I), conjugation enzymes (phase II), and key transporters. Genes showing statistically significant increases or decreases over time (see Section 4) are highlighted in dark purple (Up, positive slope) and orange (Dn, negative slope), respectively. Gray indicates that no human orthologs were found for the corresponding nonhuman genes, and white indicates nonsignificant differential expression. See See Tables S4 and S5 for normalized gene count data.

Next, we focused on the key hepatocyte-specific genes, including those involved in drug metabolism (phases I and II enzymes) and transporters (Figure 4B and Table S4). We examined both the overall level of expression (as indicated by the number of copies for each gene) and the trends in expression over time. To enable cross-species comparisons, we mapped gene names from nonhuman species to human orthologs as detailed in Section 4; therefore, human gene symbols are used throughout the article for simplicity. We performed time-trend analyses for each gene and species using normalized counts and indicate up or down trends that were significant in this figure. While expression of many of these genes showed a significant downward trend, frequently declining by 1–2 orders of magnitude, especially in monkey and dog hepatocytes, about one-third of the genes maintained their expression levels or even showed an upward trend, suggesting the preservation of cellular identity and functional integrity over time. These findings are commensurate with previous studies reporting that while long-term culture of primary hepatocytes often leads to decreased metabolism-related gene expression, specific culture strategies, such as 3D spheroids or 2D hepatocytes grown in optimized culture medium, can help maintain or enhance expression of these genes.^54,55^ Indeed, our data show that the same genes as reported previously^54,55^ to be maintained in more physiologically relevant hepatocyte cultures—*CYP1A2, CYP2D6, CYP3A4*, and *UGT1A* families—were upregulated or maintained over 14 days in the PhysioMimix LC12 model. We interpret these results as an indication that the hepatocytes did not undergo substantial dedifferentiation or stress-induced transcriptional shifts, reinforcing the suitability of the culture system for long-term applications in drug metabolism and toxicity studies. At the same time, expression of *CYP2A6, CYP2B6, CYP2C8*, and *CYP2E1* was significantly lower over 14 days, possibly reflecting selective modulation of certain metabolic pathways during long-term culture.^56^ While the observed trends highlight the nuanced, gene-specific adaptations occurring in hepatocytes maintained in PhysioMimix LC12, we found that expression of many genes is well maintained, and based on the transcriptomic data produced by this study and made publicly available, future users can determine if their targets are expressed.

### 2.3 Assessment of Species-Specific DILI in 96-Well Plates and PhysioMimix LC12 MPS

Next, we evaluated the toxic effects of several model hepatotoxic compounds in 2D and MPS models. Previous studies showed differential sensitivity among human donors and culture conditions between 2D cultures and spheroids,^56–58^ as well as species-specific effects in a different liver MPS.^39,59^ To test species specificity and compare models, we selected CPZ (human *C*_max_ = 0.5 μM^60^), BOS (human *C*_max_ = 7.4 μM^61^), and FIAU (human *C*_max_ = 1 μM^62^). Because of the limited throughput but longer-term functionality of hepatocytes in PhysioMimix LC12 MPS (Figure 3), our drug treatment experiments were conducted in a tiered approach—first, the evaluation of concentration–response in 96-well plates and, second, the time-course study in PhysioMimix LC12 MPS. Table 2 shows the concentration ranges evaluated in each series of experiments, and Figure 1 shows the duration of exposure and dosing regimens (cells were re-exposed every 48 h). In 2D experiments, cells were dosed 2 times starting on day 4 of culture with CPZ concentrations ranging from 1 to 30 μM (2–60 × human *C*_max_), BOS from 1 to 200 μM (0.1–30 × human *C*_max_), and FIAU from 1 to 100 μM (1–100 × human *C*_max_). In the PhysioMimix LC12 MPS, cells were dosed 5 times starting on day 4 of culture but only with one concentration in each experiment (Figure 1)—10 or 30 μM for CPZ; 10, 30, or 200 μM for BOS; and 10, 30, or 100 μM for FIAU. Previous studies have shown that the sensitivity of hepatotoxicity biomarkers varies across in vitro studies using hepatocytes from different species,^39^ we therefore evaluated a wide range of relevant end points. Time-course data were collected on biomarkers of liver injury (ALT, AST and LDH) and hepatocyte function (albumin and urea) in both 2D and PhysioMimix LC12 MPS, while bile acid concentrations in cell culture media and gene expression were evaluated only in PhysioMimix LC12 MPS.

**Table 2:** Drugs and Concentrations Tested in This Study

	platform	fialuridine (*C*_max_ = 1 μM)	bosentan (*C*_max_ = 7.4 μM)	chlorpromazine (*C*_max_ = 0.5 μM)	exposure duration (days)
EXP 1	PhysioMimix LC12	10 μM (10 × *C*_max_)	10 μM (1.4 × *C*_max_)	10 μM (20 × *C*_max_)	4
	96-well plate	1, 10, 30 μM (1–30 × *C*_max_)	1, 10, 30 μM (0.1–4 × *C*_max_)	1, 10, 30 μM (2–60 × *C*_max_)	4
EXP 2	PhysioMimix LC12	30 μM (30 × *C*_max_)	30 μM (4 × *C*_max_)	30 μM (60 × *C*_max_)	10
	96-well plate	1, 10, 30 μM (1–30 × *C*_max_)	1, 10, 30 μM (0.1–4 × *C*_max_)	1, 10, 30 μM (2–60 × *C*_max_)	4
EXP 3 and 4	PhysioMimix LC12	100 μM (100 × *C*_max_)	200 μM (30 × *C*_max_)	30 μM (60 × *C*_max_)	10
	96-well plate	1, 10, 30, 100 μM (1–100 × *C*_max_)	1, 10, 30, 200 μM (0.1–30 × *C*_max_)	1, 10, 30 μM (2–60 × *C*_max_)	4

Figures 5 and S6 show dose–response effects of CPZ, BOS, and FIAU in static 2D cultures on the last day of the experiment after 2 rounds of administration with data from 2 to 4 independent experiments combined. The dose–response profiles from the second day of exposure are shown in Figure S7. CPZ showed significant toxic effects in human hepatocytes based on the response of all biomarkers, although the magnitude and directionality of the response varied widely. Liver injury biomarkers (ALT and LDH) were elevated at 10 μM (20 × *C*_max_), while the albumin and urea levels in media were elevated at even lower concentrations; albumin response was biphasic, commensurate with severe cytotoxicity at 30 μM (60 × *C*_max_). In monkey hepatocytes, ALT was significantly elevated at all concentrations tested, as low as 1 μM (2 × *C*_max_), but no significant effects were observed for other biomarkers, even though they trended in the direction of hepatotoxicity. In rat hepatocytes, the only significant effect was observed for LDH at the highest concentration tested. For BOS, LDH was the most sensitive biomarker in human hepatocytes, showing a significant effect at 1 μM (0.1 × *C*_max_), with albumin, AST, and ALT effects reaching statistical significance only at the highest concentration tested (200 μM, 30 × *C*_max_). FIAU effects were much less pronounced—only albumin decreases were significant in human hepatocytes at 30 μM (30 × *C*_max_) or higher, and only ALT increased at the lowest concentration (1 μM, 1 × *C*_max_) in monkey hepatocytes, but effects of higher concentrations were not significant. No effects on these biomarkers were observed by any compound tested in dog hepatocytes. Overall, these studies in 2D hepatocyte cultures show that multiple biomarkers may need to be employed and that cell function (e.g., albumin release) needs to be tested along with the more traditional cell injury biomarkers. Overall, ALT was the most sensitive cytotoxicity-related endpoint, especially in monkey hepatocytes. Notably, while liver injury biomarkers were unaffected by FIAU in human hepatocytes, albumin secretion was significantly suppressed in a dose–response manner, again reinforcing the potential value of evaluating liver function, in this case, a potential decrease in metabolic function associated with mitochondrial toxicity of FIAU, as an indicator of the adverse effect on the liver.

**Figure 5:**
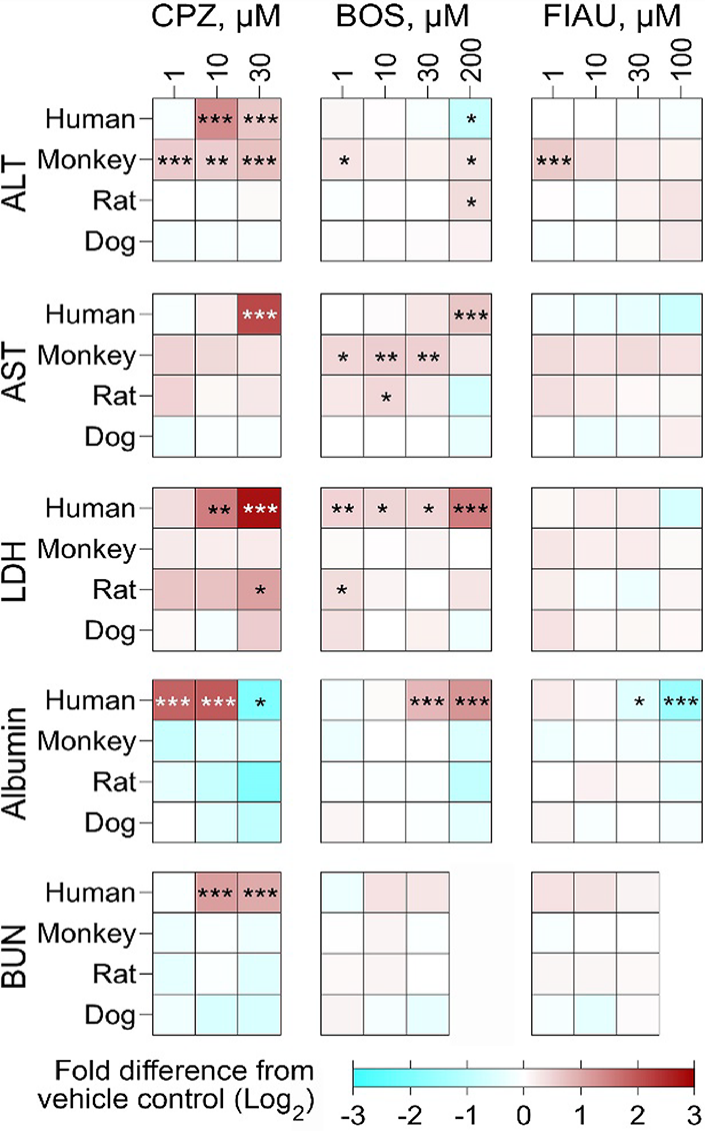
Assessment of known human DILI-causing drugs on hepatocytes' function and injury markers in 2D cultures of cross-species hepatocytes. Hepatocyte monolayers cultured in 2D format were exposed for 96 h to a range of concentrations of three reference DILI compounds: CPZ, BOS, and FIAU. Cellular injury was assessed by quantifying the release of clinical liver injury biomarkers—ALT, AST, and LDH in the culture medium. Hepatic functional integrity was evaluated by measuring secreted albumin and urea (BUN) levels. Data are presented as heatmaps representing the log_2_ fold change in biomarker levels relative to vehicle-treated controls. Each cell across the heatmap corresponds to a specific concentration of the compound tested, while each row (downward) represents different species. Red shading indicates an increase, and blue indicates a decrease relative to control levels. Each condition was tested in triplicate (*n* = 3), compiled from four independent experiments (total *n* = 3–12). Statistical significance relative to vehicle-treated controls was assessed using one-way ANOVA. Significance is indicated as follows: *p < 0.05; **p < 0.01; ***p < 0.001. See the box-and-whisker plots in Figure S6, which indicate the interquartile range and minimum–maximum values, with individual replicate data shown as dots.

More pronounced toxic effects of all three drugs were observed in the PhysioMimix LC12 MPS (Figures 6 and S8). While it is not necessarily surprising because testing was performed with only the highest dose used in 2D comparator studies and administered in repeating doses. These studies revealed additional effects and also confirmed the findings in 2D. Observed toxic effects were generally time-dependent, with the most pronounced effects observed after the third consecutive dose. Effects of CPZ (30 μM, 60 × *C*_max_) were most robust in human hepatocytes as measured by ALT, AST, LDH, and albumin; no significant change in urea levels was detected. Monkey hepatocytes showed generally similar effects and a nearly identical time course to that of human cells. Rat hepatocytes showed similar patterns, but significant findings were observed only for AST after the third dose; albumin levels showed a declining trend, but no significant effects were found on either day of testing. Dog hepatocytes were found to be responsive, unlike in 2D experiments, with ALT and LDH elevations reaching significance as early as the second dose. BOS (200 μM, 30 × *C*_max_) toxicity in human hepatocytes was most pronounced in its effects on LDH and albumin, the latter of which showed a significant decline as early as the second dose. No effects on monkey or rat hepatocytes were observed. Interestingly, an increase in ALT and a decline in urea were found in dog hepatocytes starting at the third dose. FIAU (100 μM, 100 × *C*_max_) caused a significant time-dependent decline in albumin in human and dog hepatocytes starting at the third drug dose. Declines in urea were also observed in the dog cells. Urea levels significantly increased in rat hepatocytes only at the end of the experiment. No significant effects of FIAU were detected in the monkey hepatocytes. The effects of CPZ, BOS, and FIAU following treatment with 10 and 30 μM concentrations are presented in Figures S9 and S10, respectively.

**Figure 6:**
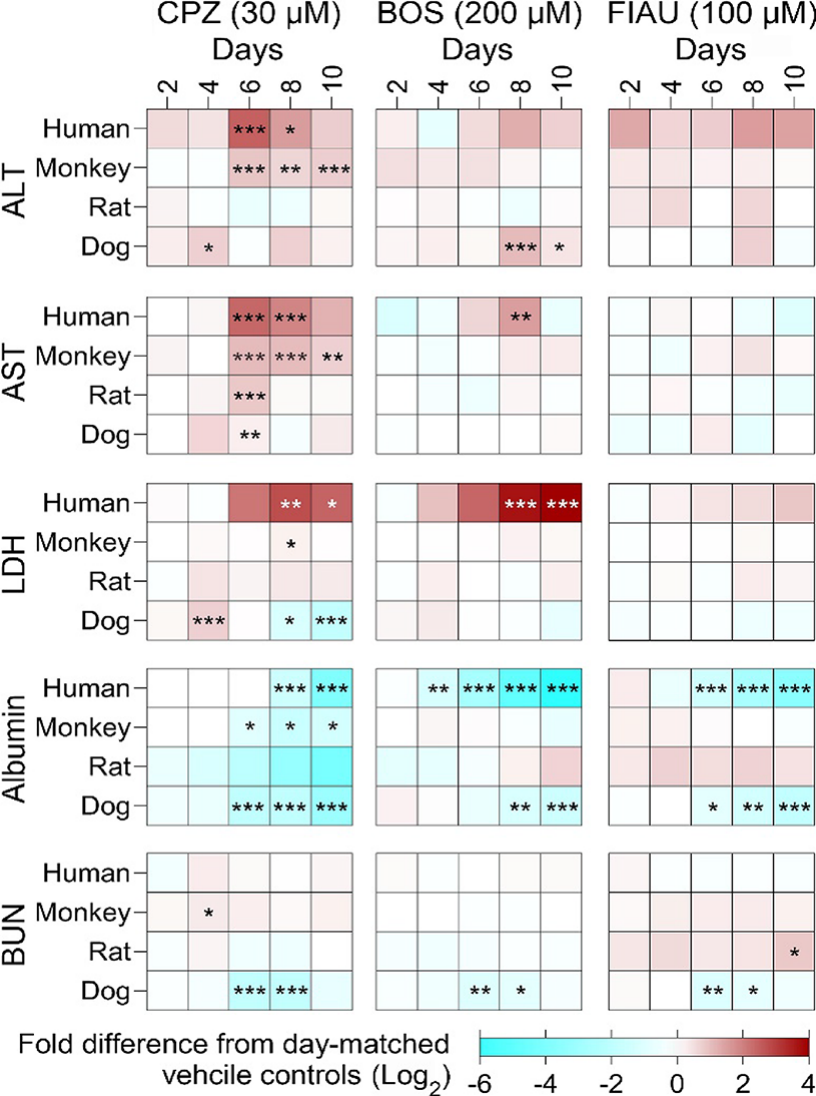
Assessment of known human DILI-causing drugs on function and injury markers of cross-species hepatocytes cultured in PhysioMimix LC12. Hepatocytes cultured in an MPS PhysioMimix LC12 were exposed to a single concentration of three reference DILI-causing drugs: CPZ (30 μM), BOS (200 μM), and FIAU (100 μM) over a 10 day period. Samples were collected while redosing on days 2, 4, 6, 8, and 10 postexposures to evaluate hepatotoxicity and functional changes. Hepatocellular injury was assessed via quantification of ALT, AST, and LDH release into the medium, while hepatic function was evaluated by measuring albumin and urea (BUN) secretion. Heatmaps display the log_2_ fold change in biomarker levels relative to vehicle-treated controls for each time point. Each cell across the heatmap corresponds to a specific day of exposure, while each row (downward) represents different species. Red shading indicates an increase, and blue indicates a decrease relative to control levels. Each condition was tested in triplicate (*n* = 3), with data compiled from two independent experiments (total *n* = 3–6). Statistical comparisons to vehicle-treated controls at each time point were performed using one-way ANOVA. Significance levels are indicated as follows: *p < 0.05; **p < 0.01; and ***p < 0.001. See the box-and-whisker plots in Figure S8, which indicate the interquartile range and minimum–maximum values, with individual replicate data shown as dots.

We also conducted power analysis of time-dependent drug effects in PhysioMimix LC12 MPS (Table S3). These analyses were performed on LDH and albumin levels as representative and most affected biomarkers (Figure 6). We found that the smallest number of replicates, generally between 2 and 5, may be sufficient to distinguish drug effects on albumin secretion after the fourth and fifth doses. Intraexperimental variability was far more considerable, requiring an even larger number of replicates, at the earlier time points.

Collectively, these experiments showed that all hepatocytes tested—from human, rat, dog, and monkey—were sensitive to CPZ-induced toxicity; however, more pronounced and consistent effects were observed in PhysioMimix LC12 MPS. These findings are consistent with a postulated conserved toxicity mechanisms of CPZ-induced liver toxicity across species, albeit few in vivo studies are available in monkeys.^20^ Similarly, BOS exhibited adverse effects on hepatocytes from all species; however, the effects were less concordant between 2D and MPS models and among species. BOS is classified as a human DILI compound^8^ and can result in liver injury and cholestasis in humans, both in vivo and in vitro. Inhibition of bile acid transporters is thought to be the primary toxicity mechanism in all species tested,^63^ albeit differential inhibition of bile acid transporters by bosentan has been suggested as a mechanism for species differences in hepatotoxicity.^15^ Previous species comparison using another liver MPS showed that BOS causes a reduction in albumin in human and dog hepatocytes,^39^ which is in agreement with our findings. The most notable results were obtained with FIAU. While previous studies found preclinical species to be largely nontranslational with respect to human liver toxicity observed in clinical trials,^11^ we found a robust response on downregulation of albumin synthesis in both human and dog hepatocytes cultured in the MPS. Even though albumin secretion gradually declined in hepatocytes cultured in both 2D and MPS in cells from all species tested (Figure 3), the ability of hepatocytes to sustain some production of albumin over 2 weeks in culture in the MPS model allows for repeat dosing and observation of the effects on this functional marker. While no cytotoxicity was observed, the decline in synthetic function of the hepatocytes (Figure 6), combined with the reports that FIAU accumulates in DNA of preclinical species after long-term administration,^64^ could have raised concerns in preclinical studies before catastrophic human trials were initiated.^10^ The decrease in albumin levels after FIAU treatment observed herein is consistent with previous findings, further supporting its role as a sensitive biomarker for in vitro DILI studies. Specifically, a dose-dependent decline in albumin secretion was previously observed in human liver organoids treated with FIAU for 10 days.^65^ Similarly, both dose- and time-dependent decreases in albumin secretion were observed on days 7 and 10 of continuous exposure in human liver spheroids and a liver MPS.^66^ In that study,^66^ other hallmarks of mitochondrial dysfunction and hepatocellular injury, such as steatosis and ATP depletion, were also reported; however, the early decline in albumin production consistently preceded cytotoxicity.

### 2.4 Mechanistic Insights into Species-Specific Drug-Mediated Hepatotoxicity in PhysioMimix LC12 MPS

Given that most informative findings of species-specific drug effects were observed in PhysioMimix LC12 MPS, we next examined effects on gene expression and bile acid synthesis in this model. Time-course gene expression data on untreated cells (Figure 4) showed that the hepatocytes from human, monkey, rat, and dog largely maintained expression of most genes over 14 days in culture. To compare drug effects, we conducted gene expression analyses on cells cultured for 14 days without treatment and cells collected at the end of 5 consecutive treatments with each tested drug (Figure 1). Figure 7 shows the analysis of differentially expressed genes (absolute log_2_ fold change > 1.5 and False discovery rate (FDR) < 0.05) in each species and treatment.

**Figure 7:**
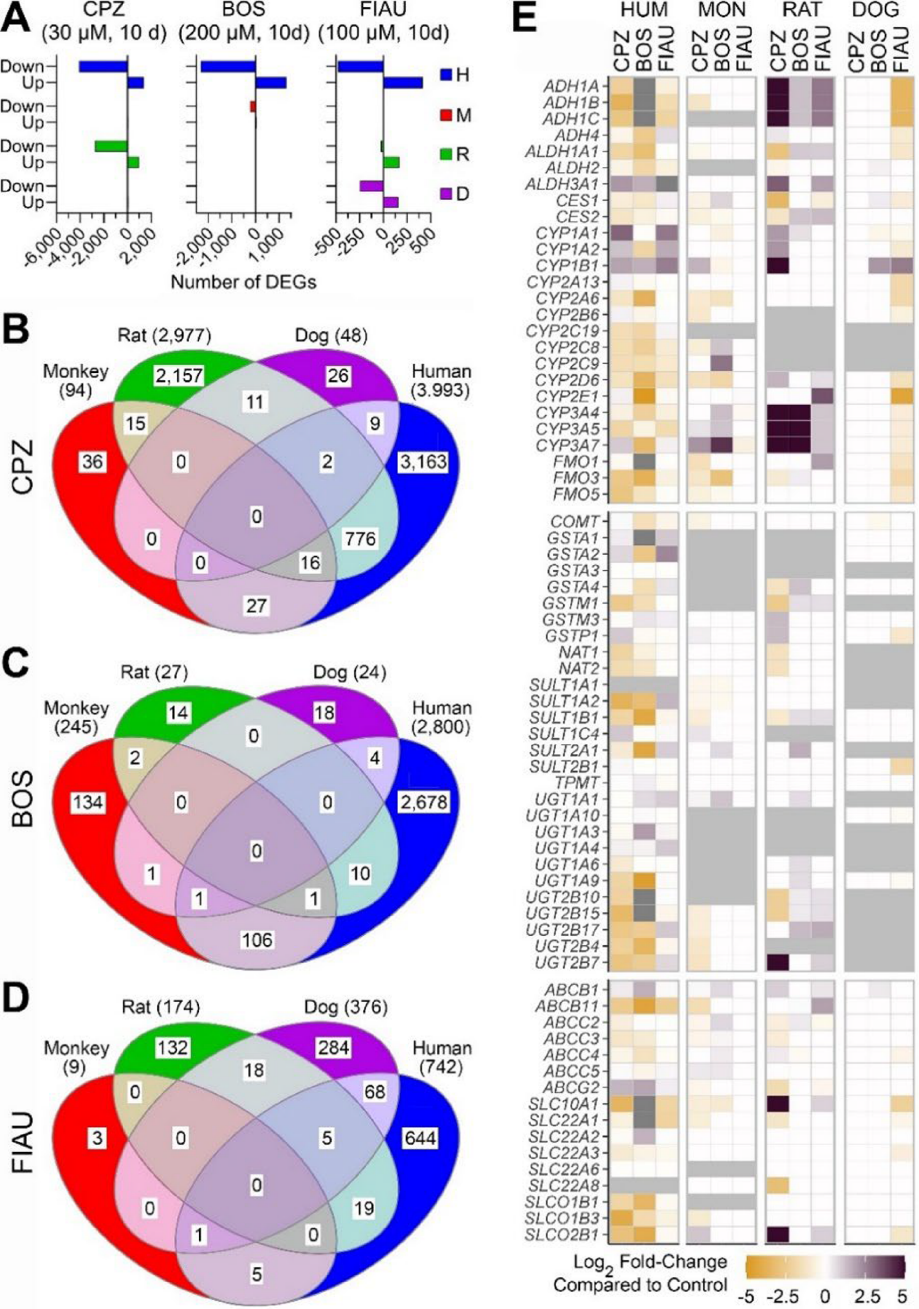
Treatment effects on hepatocyte gene expression induced by DILI compounds across species. (A) Number of differentially expressed genes (absolute log_2_ fold change > 1.5 and FDR < 0.05; see Section 4) identified within each species by comparing gene expression profiles between drug-treated and vehicle control samples. (B–D) Venn diagrams illustrating the overlap of differentially expressed orthologs across species for each treatment: (B) CPZ, (C) BOS, and (D) FIAU. (E) Heatmap showing the log_2_ fold change values of representative genes encoding for phase I enzymes (e.g., cytochrome P450 family), phase II enzymes (e.g., UGTs and SULTs), and transporters (e.g., ABC and SLC transporters) across species and treatments compared to respective vehicle controls. Dark purple indicates upregulation, orange indicates downregulation compared to vehicle controls. Gray indicates that no human orthologs were found for the corresponding nonhuman genes, and white indicates nonsignificant differential expression (FDR-adjusted *q*-value > 0.05). See Table S6 for the full list of overlapping pathways (panels B–D) and Table S7 for log_2_ fold change values for the heatmap (E).

In human hepatocytes, all three drugs elicited robust effects on gene expression (Figure 7A); most transcripts were downregulated, and the greatest effect in terms of the number of affected genes was observed with CPZ. In monkey hepatocytes, very few genes were dysregulated with some effects observed only with BOS. In rat hepatocytes, CPZ and FIAU effects were observed but less pronounced than those in human cells. In dog hepatocytes, only FIAU elicited any effect. Next, we mapped genes from nonhuman species onto the human genome and examined the overlap in each drug’s effects using the orthologs (Figure 7B–D). For CPZ (Figure 7B), the most notable overlap was found between rat and human transcriptional responses—the effect on 794 genes was conserved. These overlapping genes were strongly associated with protein and RNA metabolism pathways. Additionally, we found enriched pathways related to xenobiotic oxidation and drug metabolism. Even though the overall response in the monkey hepatocytes was muted, about half of the differentially expressed genes in the monkey overlapped with those affected in human and rat hepatocytes (Table S6). For BOS (Figure 7C), human and monkey hepatocytes were responsive—about half of the effects in the monkey cells were similar to those in human hepatocytes (Table S6). The genes overlapping between the two species were enriched in phase I and phase II metabolism pathways, as well as lipid metabolism. For FIAU (Figure 7D), the overlap in transcriptional effects was most pronounced between human and dog hepatocytes. For the genes overlapping between these species, the enriched pathways were primarily related to plasma lipoprotein remodeling (Table S6). The effects in the rat were largely unique to that species, and virtually no effect was observed in the monkey hepatocytes.

In addition, we examined treatment- and species-specific effects on the same drug metabolism genes, as shown in Figure 4. Figure 7E and Table S7 show the data for the orthologues in phase I, phase II, and transporter gene families. In human hepatocytes, the most notable induction effects were observed for *CYP1A*1/2 and *CYP1B1* by CPZ and FIAU. The former are known to be the primary metabolizing enzymes for CPZ,^67^ but liver metabolic pathways for FIAU are largely unexplored. Induction of the same genes by CPZ was also observed in rat hepatocytes, commensurate with previous findings.^68^ These genes are regulated by aryl-hydrocarbon receptor (AhR) and have been shown to be inducible;^69^ however, little is known about the role of AhR in the metabolism of these tested drugs, and further studies are needed. The main effects of CPZ and BOS comprised major downregulation across all families of drug metabolism enzymes in human hepatocytes. These effects are consistent with previously reported findings, albeit those were on a smaller number of targets.^70,71^ The most pronounced effects were on the transporters known to be involved in cholestasis, such as *ABCB11* (BSEP), *SLCO1B1,* and *SLCO2B1*. Indeed, CPZ is known to affect BSEP,^72^ and BOS is known to downregulate bile acid transporters.^73^ Collectively, transcriptional effects of BOS and CPZ confirm that they act through cholestatic mechanisms, but the effects of BOS on bile acid transporters were more pronounced in terms of the downregulation effect. Interestingly, the effects on xenobiotic metabolism genes in monkey hepatocytes were largely muted as compared to other species, concordant with the lack of other effects (Figure 6). In the rat, the most pronounced effect was the induction of several gene families by both CPZ and BOS, notably CYP3A and alcohol dehydrogenases. CPZ is known to induce *Cyp3a* in the rat liver.^74^ While little data exist on the inducibility of *Cyp3a* in rodents by BOS, this enzyme is a major cytochrome metabolizing BOS in humans;^75^ thus, the major difference in species response to BOS in terms of expression of *CYP3A* genes—a decrease in human hepatocytes vs marked induction in rodent cells—is notable because an expected in vivo response was observed in the human but not rat hepatocytes. Regarding the effects of CPZ on alcohol dehydrogenases in the rat, this finding is contrary to the report that CPZ noncompetitively inhibits ADH activity in mouse liver,^76^ thus indicating the differences between in vivo and in vitro biology. In dog hepatocytes, only FIAU showed effects on the xenobiotic enzymes. The lack of effects on xenobiotic metabolism enzymes in human, monkey, and rat hepatocytes is not surprising because the primary enzymes involved in FIAU’s metabolism are nucleoside kinases, not classic metabolism proteins.^77^ The downregulation of alcohol metabolism in dog hepatocytes, both *ADH* and *CYP2E1*, is interesting and may require further study because, currently, there is no evidence indicating that FIAU interacts with alcohol metabolism in the liver.

To interpret the overall transcriptomic effects of drugs on hepatocytes from different species using pathway analysis, several approaches were explored (Figure 8 and Table S8)—Reactome,^78^ WikiPathways,^79^ and Key Characteristics.^80^ With the Reactome database as a reference (Figure 8A), we observed concordance among human and rat transcriptional effects of CPZ on drug metabolism and pro-fibrosis pathways. For BOS, rat and human concordance in gene expression is also confirmed for a number of liver injury-relevant pathways. Interestingly, even though FIAU is not subject to classic xenobiotic metabolism, effects on phase I enzymes were shared among human, rat, and dog hepatocytes. For WikiPathways (Figure 8B), the similarities between monkey and human hepatocyte effects were more pronounced than with other approaches, even though the overall impact on the monkey cell transcriptome was small compared to that in human or rat cells. Similarly, more pathways were identified as significant for both BOS and FIAU, showing species similarities on the pathway level even though gene expression effects were of very different magnitudes. Key characteristics of known human hepatotoxicants have been recently identified^81^ and a workflow for Key Characteristics-based gene sets proposed.^80^

**Figure 8:**
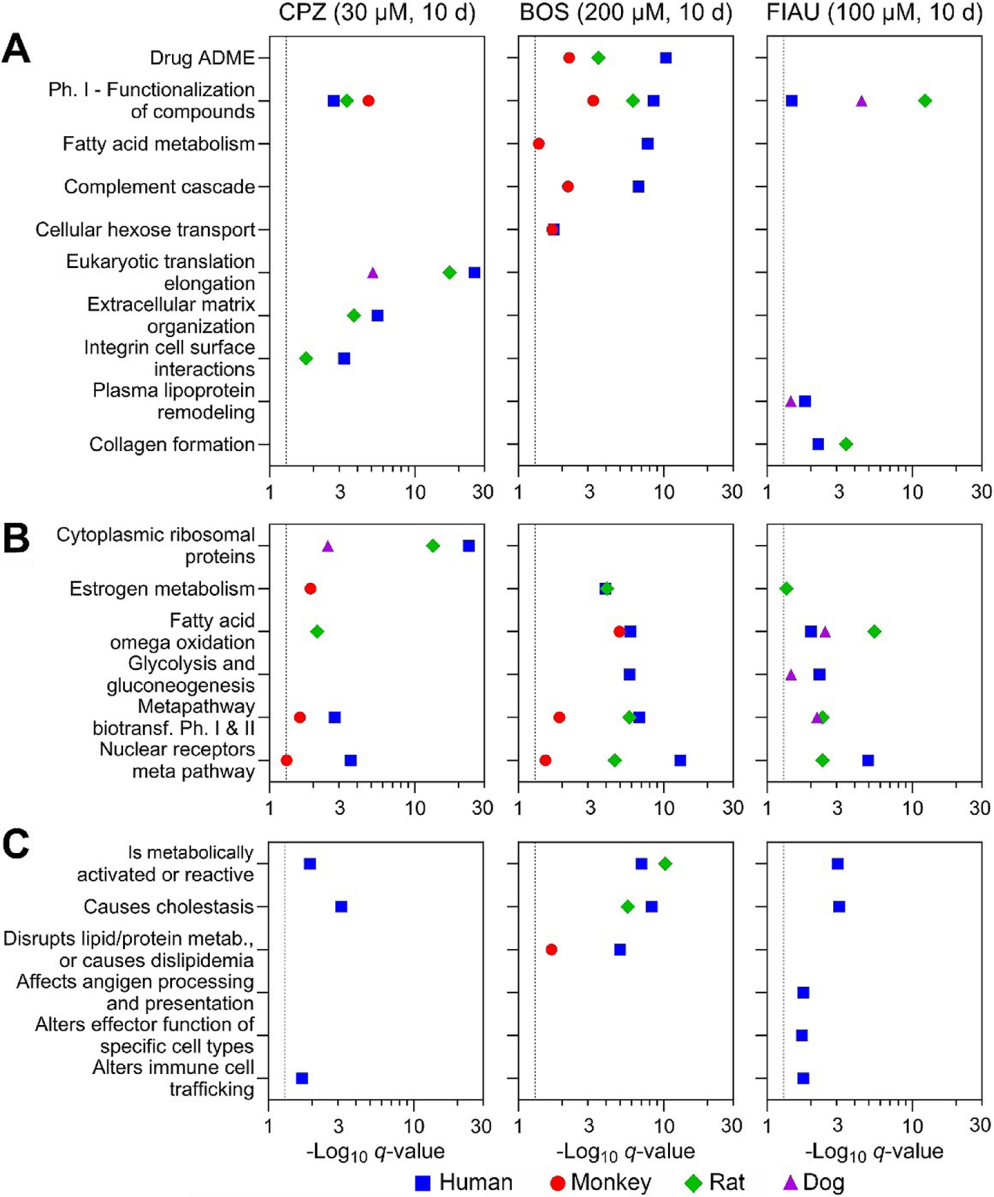
Pathway analysis of hepatocyte transcriptomic responses to DILI compounds across species using multiple databases. Scatter plots display selected enriched pathways identified from (A) Reactome, (B) WikiPathways, and (C) Key Characteristic Gene Sets, based on a significance threshold of *q-*value <0.05. Dots of different colors and shapes represent different species, as indicated in the legend at the bottom of panel C. See Table S8 for the full list of enriched pathways.

With this approach (Figure 8C), all three drugs have a clear association with the effects on metabolic activation and cholestasis, while among other species, these effects are only identifiable in the rat. Interestingly, even though our experiments did not include immune cells, several pathways related to known key characteristics of human immunotoxicants^82^ have also been identified for both CPZ and FIAU in human hepatocytes. In addition, we note that FIAU-mediated effects on fatty acid/lipid/energy metabolism pathways (Figure 8) indicate broad disruption of the mitochondrial function, bile acid homeostasis, and metabolic signaling, consistent with the clinical presentation of steatosis, lactic acidosis, and progressive liver failure reported in FIAU-treated patients.^10^ The model-omics data, combined with decreases in albumin secretion, support liver toxicity concerns for FIAU and indicate that hepatotoxicity may arise from impaired mitochondrial pathways, leading to widespread metabolic dysregulation. Additional functional assays that probe mitochondrial health, reactive oxygen species generation, bile efflux, and other mechanistic endpoints could be performed as a follow-up to the screening-type assays employed herein to further elucidate hepatotoxicity potential for additional test compounds that show similar adverse effects.

Finally, to phenotypically anchor genomic signatures of cholestasis observed for several drugs in some species-specific hepatocytes, effects reported for CPZ and BOS but not FIAU, we measured concentrations of several bile acids in cell culture media in the experiments with PhysioMimix LC12 MPS (Figures 9 and S11). We focused on the primary unmodified and glycine and taurine conjugated bile acids^83^—cholic acid (CA) and chenodeoxycholic acid (CDCA)—these are known to constitute the majority of bile acids produced by hepatocytes in culture.^84^ Figure 9A shows total bile acid levels (a sum of the two unconjugated primary bile acids CDCA and CA, together with their taurine- and glycine-conjugated derivatives such as TCDCA, GCDCA, TCA, and GCA) in cell culture media in untreated hepatocytes over time in culture, i.e., 4, 8, and 14 days. In all species, a gradual decline in bile acid release was observed, and significant effects were found in human and rat hepatocytes. Even though we found wide variability in total bile acid levels across the experiments, levels were the highest and variability smallest in monkey hepatocytes. The gradual decline in bile acid release could be indicative of the loss of the overall liver-specific synthetic function, corresponding to the observed declines in albumin and urea synthesis (Figure 3). It is also commensurate with the time-dependent decrease in expression of bile acid export transporters (Figure 4B).

**Figure 9:**
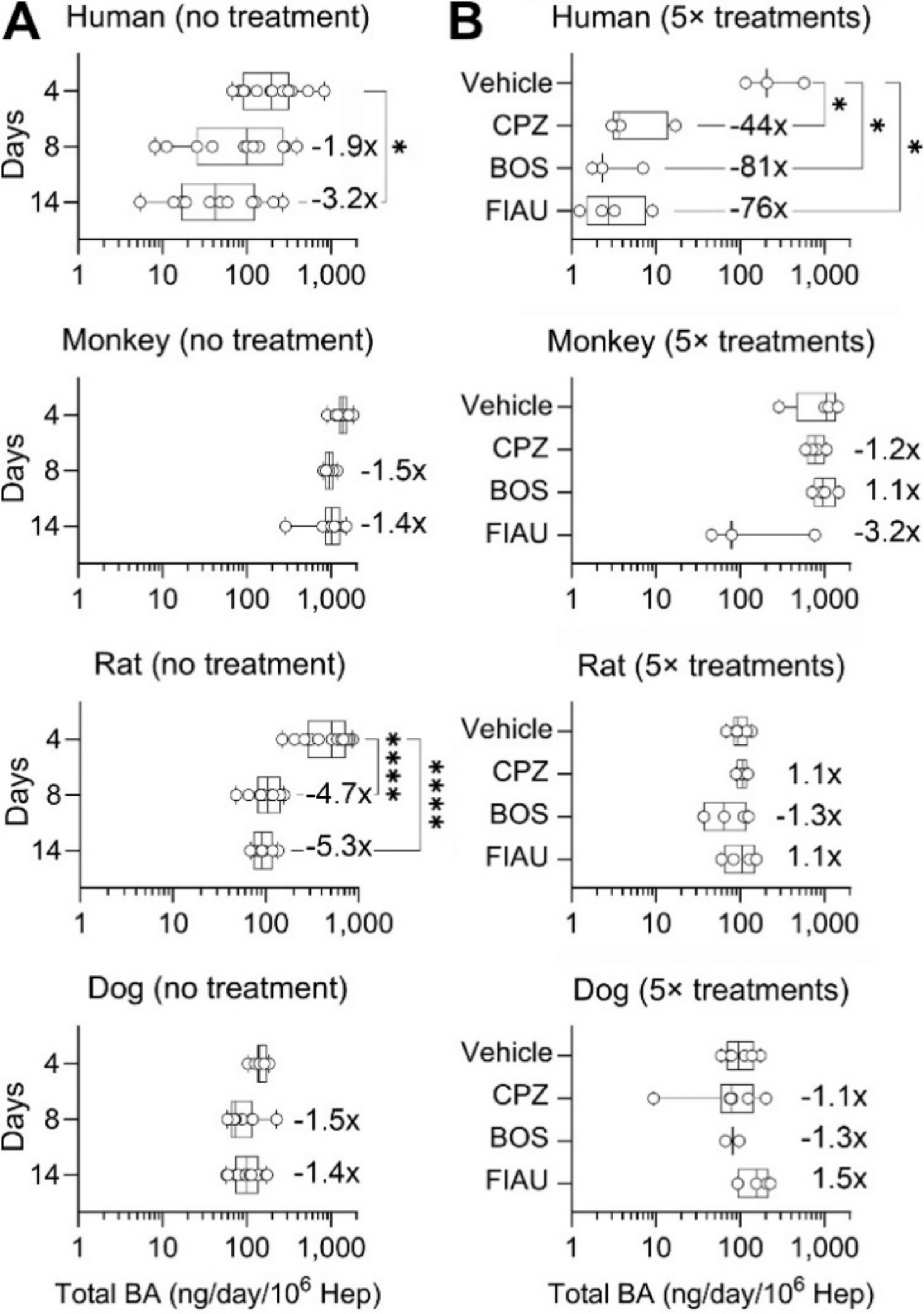
Measurement of bile acid secretion by hepatocytes cultured in PhysioMimix LC12 under basal and drug-treated conditions. Bile acid concentrations (two unconjugated primary bile acids such as CDCA and CA, together with their taurine- and glycine-conjugated derivatives, such as TCDCA, GCDCA, TCA, and GCA) were quantified in the cell culture media from species-specific hepatocytes maintained in a liver MPS to evaluate both temporal dynamics under baseline conditions and the effects of DILI-causing drugs. (A) Total bile acid levels (the sum of the bile acids listed above) were measured in the culture medium collected from species-specific hepatocytes at three time points: days 4, 8, and 14 of continuous culture. (B) To assess treatment-related effects, hepatocytes exposed to CPZ (30 μM), BOS (200 μM), and FIAU (100 μM) over a 10 day period, and total bile acid concentrations were measured on day 10. Treatment-related effects on bile acid secretion were compared to vehicle controls and visualized to highlight compound-specific changes in bile acid homeostasis. Numerical values represent the mean ± SD of *n* = 3 biological replicates per condition. Statistical comparisons were conducted using one-way ANOVA with significance defined as *p* < 0.05.

When drug-treatment-related effects were compared (Figure 9B), significant declines (44- to 81-fold) in bile acid levels in the media were observed in human hepatocytes for all three drugs. Drug effects were of far smaller magnitude and nonsignificant in nonhuman hepatocytes. The decline of bile acids in cell culture media, most often resulting from intracellular accumulation of bile acids due to transporter inhibition, indicates cholestasis—it is a known liver injury mechanism though bile acid transporter inhibition by both CPZ and BOS.^63^ These findings are also supported by the observed effects on gene expression (Figure 7E), especially for CPZ and BOS, which indicated downregulation in bile acid transporters. However, the observations of an apparent drop in bile acid release into the media in the experiments with FIAU are likely not due to the same mechanism because gene expression effects on xenobiotic transporters were not pronounced in human hepatocytes. Several key steps in the biosynthetic pathway for bile acids are directly dependent on mitochondrial enzymes and mitochondrial-derived metabolites.^85,86^ Thus, it is likely that mitochondrial damage from FIAU^13^ may indirectly interfere with hepatocyte function (including bile acid disposition). However, no studies have reported FIAU effects on bile acid levels, synthesis, transport, or receptor signaling. It is also likely that while we did not detect toxicity in human hepatocytes at concentrations similar to those shown to be cytotoxic in the same MPS,^48^ we did observe a significant decline in albumin synthesis with FIAU (Figure 6); thus, the observed effect on total bile acids could be an additional indicator of mitochondrial impairment leading to an overall decline in hepatocyte synthetic function.

## 3 Conclusions

Multiple elements of regulatory guidance that govern preclinical drug safety evaluation state that preclinical testing of any “new chemical entity” for general and reproductive toxicology studies, especially small-molecule drugs, should entail experiments in two species, a rodent and nonrodent.^87^ Therefore, there is great interest in cross-species comparisons of potential adverse effects of drugs and nonpharmaceuticals in cell-based models. Because DILI is a major concern in drug development,^88^ methods and models that can identify drug candidates that may cause DILI in humans are of high interest to both the pharmaceutical industry and the regulators.^89^ The search for clear, consistent, and translatable hepatotoxic signals in preclinical assessment, especially in cell-based models, continues with options of various complexities being available, from cultured cells to organoids/spheroids and MPS.^24^ While many published studies proposed new human liver in vitro models and suggest their utility for drug safety evaluation, few include a comparative analysis between models or across species. For example, one previous study in species-specific liver spheroids showed that six out of seven human DILI-positive compounds could be detected with cells from any preclinical species.^90^ However, there are also a number of instances where both in vitro and in vivo studies in preclinical species failed to detect DILI, with grave consequences.^10^ Thus, studies that compare and contrast both species-concordant and -discordant effects of hepatotoxicants are potentially the most informative in terms of proposing strategies for predicting human adverse effects, or derisking indications of possible liver injury in clinical trials when there was little evidence for DILI from extensive preclinical tests.^28^ One highly informative example of detecting liver toxicity in preclinical species in vitro used a multicellular flow-based liver MPS to show translational relevance for several compounds where human adversity was “missed” in preclinical studies.^39^ Species-specific drug studies in liver spheroids have also been undertaken, but the full results of these studies are yet to be publicly released.^91^ Thus, additional comparative analyses are needed to establish the potential for MPS to provide actionable information in early-stage safety evaluation. Our study focused on this goal and provided several important learnings.

Here, we highlight the utility of combining liver MPS with conventional 2D hepatocyte models in a tiered approach to assess DILI across species. The use of matched hepatocyte lots and standardized protocols between 2D and MPS studies allowed for dose selection in 2D studies and then time-course analysis in the MPS. In addition, a direct comparison of species- and platform-specific responses revealed important insights into dose-dependent and temporal toxicity patterns and should increase confidence in decision-making before in vivo safety studies are initiated. We provide a comprehensive comparative data set across four species—human, monkey, rat, and dog—extending beyond prior studies that examined two or three species. By including three preclinical species commonly used in regulatory toxicology studies alongside human hepatocytes, we offer a broader and more translationally relevant view of species-specific responses to prototypical human DILI compounds. This expanded scope strengthens the utility of the tested MPS model and our data set, including -omics data, can be used for informing species selection in future preclinical studies and refining cross-species extrapolation of hepatotoxic risk.

We found that the PhysioMimix LC12 MPS, despite the challenges with cost and throughput, represents a sensible model for longer-term in vitro studies, particularly in cases where hepatotoxicity may arise through complex or delayed mechanisms. Its ability to maintain hepatic function and morphology, albeit declining from the initial performance over 2 weeks, makes it well-suited for evaluating cumulative or later-onset toxicities that are difficult to capture in short-term 2D models, a result reported in other studies.^47,92^ The PhysioMimix LC12 MPS not only has proven effective in detecting toxicity of CPZ and BOS but also showed delayed and cumulative toxicity of FIAU in both human and dog hepatocytes—effects that were missed in 2D studies.

Another important learning is the comparative analysis of the value that multiplexing of cell injury and function biomarkers brings to in vitro liver toxicology studies. The PhysioMimix LC12 MPS allows for the measurement of both hepatocyte function (e.g., albumin, urea, and bile acid secretion) and clinically relevant DILI markers (e.g., LDH, AST, and ALT) because of the large available sampling volume. The combination of lower albumin secretion and bile acid release into the media, together with some indication of cell injury through multiple biomarkers, revealed both compound- and species-specific patterns of liver injury. However, it is very difficult to know the correct number of cells in this MPS, and it is likely that the number of viable cells declines with time and at different rates among species—the inability to image cells inside the microchannels is a considerable drawback of this model. In 2D experiments, the limitations in media volumes preclude testing of an equally comprehensive panel of phenotypes; however, inclusion of additional wells for each condition in a plate can be a potential solution. In addition, transcriptional analyses provided additional mechanistic clues of species-specific responses to hepatotoxicants, reinforcing the value of omics-based readouts in MPS.^53^ Among the various endpoints evaluated herein, gene expression emerged as both an informative and a sensitive indicator of pathway-specific effects and especially informative for differentiating among species susceptibilities. These findings suggest that incorporating transcriptomic profiling into MPS workflows can significantly enhance their translational utility.

While this study demonstrated the potential advantages of MPS in modeling long-term and species-specific hepatotoxicity, it also highlights important practical limitations. MPS platforms, such as PhysioMimix LC12, remain lower in throughput, resource-intensive, and operationally complex compared to conventional 2D culture systems. For these reasons, a tiered strategy for preclinical drug toxicity testing is required. Initial dose-range finding and screening studies should be conducted in high-throughput 2D monolayer cultures, which enable rapid testing across multiple concentrations, conditions, and species. Spheroid-based models can rival traditional 2D cultures in terms of throughput and offer a sensible intermediate-complexity-level option for the initial testing. These early-phase data can help identify key drug-induced effects and inform the selection of time points, dose, and endpoints for more focused MPS experiments. Notably, our power analysis showed that as few as 3–5 replicates could be sufficient to detect statistically meaningful drug responses in the MPS, supporting its use as a more targeted, second-tier study. However, because many mechanistic insights can already be gained from well-designed 2D experiments, MPS should be applied selectively, where added physiological relevance justifies the increased complexity and cost.

In addition, while our study provides important insights into species-specific hepatocyte functionality and drug-induced effects in static 2D cultures and a flow-based liver MPS, comparisons between these platforms are limited by the experimental design. Functional decline in 2D hepatocyte cultures^93–95^ restricted experiment duration to 8 days, while MPS allowed longer-term culture for up to 14 days. Thus, while our study highlights the improved stability and functionality of hepatocytes in the MPS, future studies should examine liver spheroids, with and without flow, as alternative higher throughput, longer-term culture conditions for comparisons with MPS. Another limitation of our study is that we did not profile proteomic or metabolomic effects of tested drugs or species and culture platform differences. These additional data will be useful for elucidating the mechanisms that may underlie the hepatotoxicity of tested chemicals, confirm that observed effects are metabolism-mediated, and provide additional clues to explain species-specific effects. Other mechanistic end points, such as evaluation of reactive oxygen species and mitochondrial toxicity assays, may provide additional clues with respect to the potential hepatotoxicity hazard of each tested compound in the follow-up studies.

Taken together, our findings support the use of MPS not as a universal replacement for either animals or simpler experimental models but as a complementary tool integrated into a pipeline for hepatotoxicity assessment. In addition, the integration of cross-species hepatocyte studies, controlled drug exposures, and longitudinal biomarker and transcriptomic profiling presents a scalable and reproducible framework for improving preclinical safety evaluation.

## 4 Materials and Methods

### 4.1 Experimental Design

This study consisted of a series of experiments designed to evaluate drug-induced liver injury across species using both microphysiological systems and conventional 2D cultures (Figure 1). All raw data and study protocols are available from the EveAnalytics database using hyperlinks listed in Table S9. Primary hepatocytes derived from human, cynomolgus monkey, rat, and dog were used in all experiments, with the same lots used across all platforms and conditions to minimize biological variability. Primary human hepatocytes (HMCPIS, lot #HU8373) were purchased from Gibco (Thermo Fisher Scientific, Waltham, MA, USA), and primary rat (Sprague–Dawley; M00005-P, lot #FIM), monkey (Cynomolgus; M003055-P, lot #JCW), and dog (Beagle Dog; M002055-P, lot #BTQ) hepatocytes were purchased from BioIVT (Baltimore, USA).

Hepatocytes were cultured in the PhysioMimix LC12 MPS (CNBio, Cambridge, UK) and in parallel 2D monolayers in collagen I-coated 96-well plates. In the MPS system, cells were seeded at an initial density of 500,000–600,000 cells per chip and maintained under continuous perfusion (1 μL/s). In 2D plates, hepatocytes were seeded at 70,000–100,000 cells/well. Both systems were incubated at 37 °C and 5% CO_2_, and media were refreshed every 1–2 days. Effluent samples were collected regularly for biochemical assays. The day of seeding was designated as day 0. The overall culture duration in LC12 was 14 days, while 2D cultures were maintained for 8 days due to previously reported loss of differentiated hepatocyte functions in 2D cultures.^93–95^ Drug treatments with CPZ (cat. no. C8138, Sigma-Aldrich), BOS (cat. no. SML1265, Sigma-Aldrich), and FIAU (cat. no. SML0632, Sigma-Aldrich)—three well-characterized DILI reference compounds—were initiated on day 4 of culture and continued with media replenishment every other day. Biochemical markers of hepatocyte viability and function were assessed in collected media, including albumin and urea secretion, and clinical liver injury markers such as LDH, ALT, and AST.

A total of five experiments were conducted, each following a specific dosing and duration regimen, as summarized in Figure 1. Experiment 1 was a short-term, low-dose exposure study that lasted 8 days. LC12 cultures were treated with a single drug concentration of 10 μM. In parallel, 2D cultures were exposed to a concentration range of 1, 10, and 30 μM. Experiment 2 was a long-term, middose study with a total culture duration of 14 days and a 10 day treatment period. LC12 received 30 μM of each drug, while 2D cultures were again treated with 1–30 μM concentrations. Experiments 3 and 4 were long-term, high-dose studies, also lasting 14 days with 10 days of treatment. In MPS, the dose levels were CPZ (30 μM), BOS (200 μM), and FIAU (100 μM). In 2D, the same compounds were tested at 1, 10, and 30 μM, and the higher doses of BOS (200 μM) and FIAU (100 μM) were added for comparison. Experiment 5 was a drug-free time-course study in the LC12 system, conducted over 14 days to evaluate the temporal dynamics of hepatocyte gene expression in the absence of a pharmacological challenge. Cell samples were collected on days 4, 8, and 14 for transcriptomic profiling. In addition, scaffold-resident hepatocytes were imaged using bright-field microscopy to monitor morphological changes over time. Across all experiments, replicates were used to assess the reproducibility and variability of the biochemical measurements. Both intra- and interexperimental variability were analyzed, and coefficients of variation (CV) were calculated for key functional readouts. These were compared against a benchmark CV of ≤ 30%, consistent with the quality criteria for liver MPS systems. Power analyses were also conducted to estimate the minimum number of replicates required to detect statistically significant differences in drug effects and hepatic function over time.

### 4.2 Cell Culture Methods and Drug Treatment

A PhysioMimix LC12 plate contains 12 individual devices/chips. Each chip contains a media reservoir and a culture well consisting of a plastic retaining ring that holds a scaffold and filter in place. Cells were seeded onto and cultured within the scaffold as detailed previously.^40^ The plate was attached to a pneumatic docking station, which has micropumps that produce recirculating media flow, which is adjustable using the PhysioMimix LC12 controller. The PhysioMimix LC 12 was primed with species-specific hepatocytes cell culture media 2 days before the cell seeding. On the day of cell seeding, primary human hepatocytes (PHHs) were thawed in cryopreserved hepatocyte recovery medium (CM7000, Thermo Fisher, USA), collected by centrifugation (100 × *g*, 10 min), and resuspended in William’s E media (A1217601, ThermoFisher) supplemented with 3.6% cocktail A, 5% FBS, 10 nM dexamethasone (CM3000, ThermoFisher). Primary monkey, rat, and dog hepatocytes were thawed directly into the INVITROGRO CP medium (BioIVT, USA) types Z990003, Z990028, and Z990025, respectively; the composition of the species-specific medium is proprietary and has been optimized by the manufacturer for each species. The cells were then seeded into the PhysioMimix LC12 scaffold at a seeding density of 500,000–600,000 cells/chip.

For the 2D static model, hepatocytes were seeded at a density of 70,000–100,000 cells per well in 200 μg/mL collagen I (CB354249, Corning, Inc.) precoated 96-well plates. Both the plates (96-well plates and LC12) were incubated at 37 °C, 5% CO_2_, and for PhysioMimix LC 12 the flow was initiated at 1 μL/s. After overnight incubation the hepatocytes media was changed to maintenance media which consists of William’s E media with supplements (4% cocktail B, 1 nM dexamethasone (CM4000, ThermoFisher) for PHH and INVITROGRO CP medium containing TORPEDO antibiotic mix (Z990008, 1 mL per 45 mL media) for monkey and dog and TORPEDO Rodent antibiotic mix (Z990027) for rat hepatocytes.

The day when hepatocytes were seeded into PhysioMimix LC12 plates or 96-well plates was defined as day 0 (D0), and the media were refreshed every 1–2 days. On day 4, the cells were exposed repeatedly (every other day) to CPZ, BOS, and FIAU until day 14 of culture in PhysioMimix LC12. The same treatment was done in a static 96-well plate until day 8. A 100X stock solution of each chemical was prepared in DMSO, and the desired doses were achieved through serial dilution while maintaining the DMSO concentration at no more than 0.1%. In all experiments, 0.1% DMSO was included as a vehicle control. During each media exchange, the spent media was collected from the wells, centrifuged to remove cellular debris, and stored at −80 °C until further analysis.

### 4.3 Biochemical Analyses

Albumin levels were quantified by using ELISA assays. Human albumin was measured using an E88–129 kit purchased from Bethyl Laboratories (Montgomery, TX). Monkey (cat. no. E-85AL), rat (cat. no. E-25AL), and dog (cat. no. E-40AL) albumin levels were quantified using kits from Immunology Consultants Laboratory, Inc. Urea secretion was quantified using either the EIABUN kit from ThermoFisher or the QuantiChrom Urea Assay Kit (DIUR-100) from BioAssay Systems (Hayward, CA). LDH release, AST, ALT activity was measured using a colorimetric method. LDH assay kit (Ab102526) was purchased from Abcam (Waltham, MA). AST activity kit (cat. no. 701640) was obtained from Cayman Chemical Company (Ann Arbor, Michigan), and ALT activity kit (ab24103) was purchased from Abcam. All assays were performed according to the protocols provided by the respective vendors.

### 4.4 Bile Acid Measurements

Bile acids were extracted from the cell culture media using acetonitrile containing an internal standard (glycolithocholic acid (2,2,4,4-D_4_), DLM-9556-C; Cambridge Isotopes, Tewksbury, MA). Briefly, 300 μL of media was added to an equal volume of acetonitrile with internal standards. The solution was briefly vortexed and centrifuged at room temperature at 12,000 rpm for 10 min. The supernatant collected was transferred to a new microcentrifuge tube and lyophilized to using a Christ Alpha 1–2 LD Plus freeze-dryer and then reconstituted with methanol (20 μL). The extracts were stored at −20◦C until further analysis. The two unconjugated primary bile acids (CDCA and CA) along with their taurine and glycine conjugates (GCDCA, TCDCA, GCA, TCA) were quantified using a triple quadrupole LC-MS/MS system, model LCMS-8045 (Shimadzu Corporation, Kawasaki, Japan). Samples were separated on a Kinetex C18 column (1.7 μm × 100A × 50 mm × 2.1 mm, Phenomenex 00B-4475-A) with a C18 2.1 mm security guard (Phenomenex AJ0-8782) precolumn using an ultrahigh performance liquid chromatography system with gradient elution. Mobile phase A was Milli-Q water with 0.01% formic acid and mobile phase B was methanol/acetonitrile (50% v/v). Data acquisition and processing were conducted using LabSolutions software (Shimadzu). The limit of detection (LOD) for each BA was defined as the lowest concentration yielding a signal-to-noise ratio greater than 5, while the limit of quantification (LOQ) was set at the lowest concentration with a signal-to-noise ratio above 10.^96^ The two unconjugated primary bile acids CDCA and CA, together with their taurine- and glycine-conjugated derivatives such as TCDCA, GCDCA, TCA, and GCA, were quantified. The concentrations of these six bile acids were summed to derive the total bile acid value for each sample.

### 4.5 RNA Isolation, Library Preparation, and Sequencing

Total RNA was extracted using TRIzol reagent (Invitrogen, Carlsbad, CA) and the Direct-zol RNA Miniprep (Zymo, Irvine, CA) following the manufacturer’s protocol with slight modifications detailed here. MPS scaffolds from each experiment were preserved in RNAlater at −80 °C until RNA extraction. On the day of extraction, samples were thawed at room temperature, and each scaffold was cut into two pieces before being transferred to a 1.5 mL microcentrifuge tube. Next, 700 μL of TRIzol reagent was added to the samples, which were then incubated at room temperature for 20 min. During incubation, the samples were vortexed for 30 s every 5 min to ensure complete lysis. The scaffolds were removed from the microcentrifuge, and the RNA was precipitated using an equal volume of 100% molecular grade ethanol and then transferred to a Zymo-Spin IICR column. The remainder of the extraction was performed according to the standard protocol. RNA was eluted in 60 μL of DNAase/RNase-free water. Total RNA concentration and yields were determined using the Nanodrop 8000 instrument (Thermo Fisher Scientific), and A260:A280 values were obtained for qualitative assessment. The RNA samples were then stored at −80 °C until library preparation and sequencing.

### 4.6 Library Preparation and Sequencing

96 samples were submitted for sequencing in 1 plate. Initial quality control was performed on 11 randomly selected test samples per plate. RNA batch purity was determined based on sample absorbance in the 230–280 nm range as measured by Unchained Laboratories (Pleasanton, CA) Lunatic Plates, read on a PerkinElmer (Waltham, MA) DropletQuant spectrophotometer. The same test samples were subjected to parallel capillary electrophoresis on an Advanced Analytical (now Agilent, Santa Clara, CA) 5200 Fragment Analyzer System to assess the RNA integrity. Total RNA concentration was estimated using absorbance at 260 nm on a DropletQuant Spectrophotometer. mRNA was isolated from 150 ng of total RNA using a Nextflex Poly-A Selection kit. Unique dual-indexed cDNA libraries were prepared on a Sciclone NGSx liquid handler, using a Nextflex Rapid Directional RNA 2.0 kit miniaturized to a 2/5 reaction volume. Optimal PCR cycle counts were empirically determined to be 17 by cycling a pool of samples to the plateau phase on a real-time PCR machine. The concentration of libraries was determined using a Molecular Devices (San Jose, CA) Spectramax M2 fluorescent plate reader and Thermo Fisher Scientific (Waltham, MA) Picogreen Assay. Libraries were then pooled by equal mass using a Janus Mini liquid handler. The pooled library was sequenced on an Illumina NovaSeq X+ (San Diego, CA) using a two-lane, paired-end 2 × 150 bp configuration on a 1.5B flowcell. Sequence cluster identification, quality prefiltering, base calling, and uncertainty assessment were done in real time using Illumina’s NCS 1.0.2 and RFV 1.0.2 software with default parameter settings. Sequencer basecall files (BCL) were demultiplexed and formatted into FASTQ files using bcl2fastq 2 2.19.0, yielding 1.9 billion demultiplexed clusters, with an average of 20 million clusters per sample. Data quality was evaluated using FastQC 0.11.9 and MultiQC v1.9. The FASTQ files were uploaded to Gene Expression Omnibus (GEO; accession number GSE300171; reviewer token orydykkezfqtxcj).

### 4.7 Transcriptomic Data Analysis

Paired-end FASTQ files (R1 and R2) were generated per sample by combining reads from both sequencing lanes. The concatenated FASTQ files were next preprocessed using fastp version 0.23.2 ^103^ with the following steps: (1) automatic detection of adapter sequences for paired-end reads, (2) trimming of homopolymer stretches, and (3) removal of bases with Phred quality scores below 20. Reads from dog, monkey, human, and rat were mapped to the ROS_Cfam_1.0, Macaca_fascicularis_6.0, GRCh38, and mRatBN7 genome assemblies, respectively, were aligned to the species-specific genome assembly using STAR version 2.7.10b.^97^ Aligned reads were sorted with SAMtools version 1.14,^98^ and raw count data were generated using HTSeq version 2.0.1 ^99^

Raw count data were filtered using the following quality control steps. Samples with fewer than 1 million total counts or with uniquely mapped reads below 50% were excluded from further analysis. Three samples—one human sample from the day 4 time point and two rat samples from the CPZ treatment group—were removed for failing these criteria. The remaining samples were assessed for potential outliers using principal component analysis (PCA) and cross-replicate correlation analysis; no samples were excluded in this step. For transcript-level quality control, transcripts that were not expressed across the full sample set for each species were removed. Within-species normalization of count data was performed using the Bioconductor package DESeq2.^100^ For cross-species gene-level comparisons, Ensembl IDs for human were converted to HGNC symbols, and Ensembl IDs from nonhuman species were mapped to their human orthologs using the Bioconductor package biomaRt (version 2.64.0).

To assess temporal effects, a heatmap of normalized counts for all genes with human orthologs was generated using the pheatmap R package, with default clustering and distance settings. Temporal trends in gene expression were examined for a selected set of Phase I enzymes, Phase II enzymes, and transporter genes using the lm­() function in R. The direction of the trend was recorded, and adjusted p-values from the trend tests were filtered using an FDR threshold of <0.05. To evaluate chemical treatment effects, treated samples were compared to corresponding untreated controls within each species using the DESeq2 R package.^100^ Log_2_ fold change values were shrunk using the lfcShrink­() function (type = “apeglm”). Differentially expressed genes (DEGs) for each treatment in each species were identified using an FDR-adjusted *q*-value <0.05 and an absolute log_2_ fold change >1.5. Pathway analysis was performed on DEGs to explore potential mechanisms underlying treatment effects. Enrichment analysis for Reactome and WikiPathways was conducted using the ReactomePA^101^ clusterProfiler R packages,^102^ respectively. To support hazard characterization, a gene set enrichment workflow based on the Key Characteristics^80^ was applied. The gene set background for all pathway analyses consisted of the interrogated genes retained after low-count filtering for each species (see the above for selection criteria).

### 4.8 Statistical Analysis

Statistical analysis was conducted using GraphPad Prism 9.2 (San Diego, CA). For comparison, one-way analysis of variance (ANOVA) followed by Tukey’s tests was used. For multiple comparisons, two-way ANOVA with Sidak’s correction was used. Power analyses for sample size needed to reach statistical significance with 80% statistical power were estimated using (i) the two-sided paired *t*-tests and (ii) two-sided two-sample *t*-tests with different variances for paired and independent samples, respectively. MATLAB (v. R2018a), SAS (v. 9.4), and PASS (v. 15) software packages were used to estimate the effect size and calculate the required sample size. Data in graphs are presented as mean ± SD and all statistical analysis and graphs were generated using GraphPad Prism. Significant differences are denoted by asterisks in associated graphs as follows: *p < 0.05, **p < 0.01, ***p < 0.001, and **** *p* < 0.0001.

## Supplementary Material





## Notes

Table S1. Sample size estimates for observing time-related differences in albumin and urea levels in experiments with human (H), monkey (M), rat (R), and dog (D) hepatocytes cultured in 96-well plates or PhysioMimix LC12 MPS. Shown are the number of replicates (wells of devices) needed for detecting significant (*p* < 0.05 with 80% power) differences between time points. Table S2. The number of samples needed to observe a significant difference (with 80% power and significance level 0.05 based on the paired Cohen’s D test between time points for LDH release into the media. Data is shown separately for human (H), monkey (M), rat (R), and dog (D) hepatocytes cultured in 96-well plates or PhysioMimix LC12 MPS. Table S3. Sample size estimates for observing time-related differences in compounds-induced toxicity in experiments with PhysioMimix LC12 seeded with primary hepatocytes from human, monkey, rat, and dog. Shown are the number of replicate chips needed for detecting significant (*p* < 0.05 with 80% power) differences between time points or in a trend test for LDH and albumin end points. Abbreviations: CPZ, chlorpromazine; BOS, bosentan; FIAU, fialuridine; H, human; M, monkey; R, rat; D, dog. Asterisks indicate instances where no time-dependent trend was observed. Table S9. List of studies used in the figures with links to the raw data in EveAnalytics Database. Figure S1. Comparison of functional differences (Albumin, left, and Urea, right, secretion) of hepatocytes in 2D static culture (96-well plates) and MPS PhysioMimix LC12 and on day 4 of culture. Data are presented as box plots representing the 10th to 90th percentiles, *p* < 0.001 (***), *n* = 5 to 12 from 5 independent experiments. The corresponding p-values for Albumin in other species are as follows: monkey – o.23; rat – 0.39, and dog – > 0.99. Figure S2. Intraexperimental variability (expressed as CV) of functional parameters albumin (A) and urea (B) when using the PhysioMimix LC12 and 96-well plates. Colors indicate data from separate experiments (see Figure 1 and Section 4 for details). The line is a nonlinear fit (third order polynomial) to the data. Dotted horizontal line indicates 30% CV. Figure S3. (A) Time course of LDH release as a stress marker in 2D static culture (96-well plates, left panel) and MPS PhysioMimix LC12 (right panel) for hepatocytes from different species (see labels). The box plot shows the median, interquartile range (IQR), and 10th–90th percentiles. (B) Intraexperimental variability (expressed as CV) of stress marker LDH when using the 2D static culture (96-well plates) and PhysioMimix LC12. Colors indicate data from separate experiments (see Figure 1 and Section 4 for details). The line is a nonlinear fit (third order polynomial) to the data. Dotted horizontal line indicates 30% CV. Figure S4. (A) Time-course analysis of liver toxicity biomarkers (AST) in 2D static culture (96-well plates, left panels) and MPS PhysioMimix LC12 system (right panels) for hepatocytes from different species (see labels). The box plots represent the median, interquartile range (IQR), and 10th–90th percentiles, illustrating the distribution and variability of AST levels across different experimental conditions. (B) Intraexperimental variability, expressed as the coefficient of variation (CV) in both the 2D static culture (96-well plates) and the PhysioMimix LC12. Colors indicate data from separate experiments (see Figure 1 and Section 4 for details). The line is a nonlinear fit (third order polynomial) to the data. Dotted horizontal line indicates 30% CV. Figure S5. (A) Time-course analysis of liver toxicity biomarkers (ALT) in 2D static culture (96-well plates, left panels) and MPS PhysioMimix LC12 (right panels) for hepatocytes from different species (see labels). The box plot represents the median, interquartile range (IQR), and 10th–90th percentiles. (B) Intraexperimental variability, expressed as the coefficient of variation (CV), was in both the 2D static culture and the PhysioMimix LC12. Colors indicate data from separate experiments (see Figure 1 and Section 4 for details). The line is a nonlinear fit (third-order polynomial) to the data. Dotted horizontal line indicates 30% CV. Figure S6. Assessment of known DILI-causing drugs on hepatocytes function and injury markers in 2D cultures. Shown are box-and-whiskers plots indicating interquartile range and min-max values with individual well data shown as dots. Data for hepatocytes from humans (blue), monkeys (red), rats (green) and dogs (purple) are shown for CPZ (left), BOS (middle) and FIAU (right). Concentration–response (in μM) is shown, and data are from the last day of exposure. See the heatmap showing these data in Figure 5 of the manuscript. Figure S7. Assessment of known DILI-causing drugs on hepatocytes function and injury markers in 2D cultures on 2nd day of exposure. For LDH, shown are box-and-whiskers plots indicating interquartile range and min-max values with individual MPS data shown as dots. Data for hepatocytes from humans (blue), monkeys (red), rats (green) and dogs (purple) are shown for CPZ (left), BOS (middle) and FIAU (right). For other biomarkers, individual values are shown, and the horizontal line indicates the median value. Figure S8. Assessment of known DILI-causing drugs on hepatocytes function and injury markers in PhysioMimix LC12. Shown are box-and-whiskers plots indicating interquartile range and min-max values with individual MPS data shown as dots. Data for hepatocytes from humans (blue), monkeys (red), rats (green) and dogs (purple) are shown for CPZ (left), BOS (middle) and FIAU (right). Time course (days of treatment) is shown for repeat-dose treatments. See the heatmap showing these data in Figure 6 of the manuscript. Figure S9. Assessment of known DILI-causing drugs on hepatocytes function and injury markers in PhysioMimix LC12 treatment with 10 μM of CPZ, BOS and FIU for 4 days. Shown are box-and-whiskers plots indicating interquartile range and min-max values with individual MPS data shown as dots. Data for hepatocytes from humans (blue), monkeys (red), rats (green) and dogs (purple) are shown for CPZ (left), BOS (middle) and FIAU (right). Time course (days of treatment) is shown for repeat-dose treatments. Figure S10. Assessment of known DILI-causing drugs on hepatocytes function and injury markers in PhysioMimix LC12 treatment with 30 μM of CPZ, BOS and FIAU for 10 days. Shown are box-and-whiskers plots indicating interquartile range and min-max values with individual MPS data shown as dots. Data for hepatocytes from humans (blue), monkeys (red), rats (green) and dogs (purple) are shown for CPZ (left), BOS (middle) and FIAU (right). Time course (days of treatment) is shown for repeat-dose treatments. Figure S11. Time-course analysis of bile acid secreted by untreated cells at 4, 8, and 14 days of the experiment using the PhysioMimix LC12 system. Data is presented as box-and-whiskers plots from 3 to 6 replicates samples indicating interquartile range and min-max values with individual MPS data shown as dots. Abbreviations: CDCA, Chenodeoxycholic acid; GCDCA, Glycochenodeoxycholic acid; TCDCA, Taurochenodeoxycholic acid; CA, Cholic acid; GCA, Glycocholic acid; TCA, taurocholic acid. (PDF)
Table S4. Normalized gene count data (Supplemental table for Figure 4A). Table S5. Normalized gene count data (Supplemental table for Figure 4B). Table S6. Full list of overlapping pathways (Supplemental table for Figure 7B–D). Table S7. Log_2_-fold change values of ADME genes (Supplemental table for Figure 7E). Table S8. Full list of enriched pathways after drug treatment (Supplemental table for Figure 8). (XLSX)

## Abbreviations

2D: two-dimensional
ADME: absorption, distribution, metabolism, and excretion
AhR: aryl-hydrocarbon receptor
ALT: alanine aminotransferase
ANOVA: analysis of variance
AST: aspartate aminotransferase
BOS: bosentan
BSEP: bile salt export pump
cDNA: complementary DNA
CPZ: chlorpromazine
CV: coefficients of variation
DEG: differentially expressed genes
DILI: drug-induced liver injury
DMSO: dimethyl sulfoxide
ELISA: enzyme linked immunosorbent assay
EMA: European Medicines Agency
FBS: fetal bovine serum
FDA: Food and Drug Administration
FDR: false discovery rate
FIAU: fialuridine
iPSC: induced pluripotent stem cell
LC-MS/MS: liquid chromatography-tandem mass spectrometry
LDH: lactate dehydrogenase
MPS: microphysiological system
PCR: polymerase chain reaction
PHH: primary human hepatocyte
RNA: ribonucleic acid
SD: standard deviation

## References

[1] Avigan M. I., Bjornsson E. S., Pasanen M., Cooper C., Andrade R. J., Watkins P. B., Lewis J. H., Merz M. (2014). Liver safety assessment:
required data elements and best practices for data collection and
standardization in clinical trials. Drug Saf..

[2] European
Association for the Study of the Liver (2019). EASL Clinical Practice Guidelines: Drug-induced liver
injury. J. Hepatol..

[3] US FDA . Roadmap to Reducing Animal Testing in Preclinical Safety Studies; US FDA: Washington, DC, 2025. 10.1002/cpdd.70046 41766281

[4] Kullak-Ublick G. A., Andrade R. J., Merz M., End P., Benesic A., Gerbes A. L., Aithal G. P. (2017). Drug-induced liver injury: recent
advances in diagnosis and risk assessment. Gut.

[5] Lee W. M. (2013). Drug-induced
acute liver failure. Clin. Liver Dis..

[6] Andrade R. J., Chalasani N., Bjornsson E. S., Suzuki A., Kullak-Ublick G. A., Watkins P. B., Devarbhavi H., Merz M., Lucena M. I., Kaplowitz N., Aithal G. P. (2019). Drug-induced liver injury. Nat. Rev. Dis. Primers.

[7] Olson H., Betton G., Robinson D., Thomas K., Monro A., Kolaja G., Lilly P., Sanders J., Sipes G., Bracken W., Dorato M., Van Deun K., Smith P., Berger B., Heller A. (2000). Concordance
of the toxicity of pharmaceuticals
in humans and in animals. Regul. Toxicol. Pharmacol..

[8] Chen M., Suzuki A., Thakkar S., Yu K., Hu C., Tong W. (2016). DILIrank: the largest reference drug
list ranked by the risk for
developing drug-induced liver injury in humans. Drug Discov Today.

[9] Kaplowitz N. (2005). Idiosyncratic
drug hepatotoxicity. Nat. Rev. Drug Discov.

[10] McKenzie R., Fried M. W., Sallie R., Conjeevaram H., Di Bisceglie A. M., Park Y., Savarese B., Kleiner D., Tsokos M., Luciano C. (1995). Hepatic
failure and
lactic acidosis due to fialuridine (FIAU), an investigational nucleoside
analogue for chronic hepatitis B. N Engl J.
Med..

[11] Institute of Medicine. Review of the Fialuridine (FIAU) Clinical Trials. In Review of the Fialuridine (FIAU) Clinical Trials, Manning, F. J. ; Swartz, M. , Eds.; National Academies Press (US): Washington (DC), 1995. 25121268

[12] Kleiner D.
E., Gaffey M. J., Sallie R., Tsokos M., Nichols L., McKenzie R., Straus S. E., Hoofnagle J. H. (1997). Histopathologic
changes associated with fialuridine hepatotoxicity. Mod. Pathol..

[13] Honkoop P., Scholte H. R., de Man R. A., Schalm S. W. (1997). Mitochondrial injury.
Lessons from the fialuridine trial. Drug Saf.

[14] Fattinger K., Funk C., Pantze M., Weber C., Reichen J., Stieger B., Meier P. J. (2001). The endothelin
antagonist bosentan
inhibits the canalicular bile salt export pump: a potential mechanism
for hepatic adverse reactions. Clin Pharmacol
Ther.

[15] Leslie E. M., Watkins P. B., Kim R. B., Brouwer K. L. (2007). Differential inhibition
of rat and human Na+-dependent taurocholate cotransporting polypeptide
(NTCP/SLC10A1)­by bosentan: a mechanism for species differences in
hepatotoxicity. J. Pharmacol Exp Ther.

[16] US FDA. Drug Approval Package: Tracleer (Bosentan) Tables; Company: Actelion, Ltd; Application No: 21–290; US Food and Drug Administration: Silver Spring, MD, 2001.

[17] Mueller S. O., Guillouzo A., Hewitt P. G., Richert L. (2015). Drug biokinetic and
toxicity assessments in rat and human primary hepatocytes and HepaRG
cells within the EU-funded Predict-IV project. Toxicol In Vitro.

[18] Larrey, D. ; Ripault, M. -P. Hepatotoxicity of Psychotropic Drugs and Drugs of Abuse. In Drug-Induced Liver Disease, Kaplowitz, N. ; DeLeve, L. D. , Eds.; Academic Press: Boston, 2013; pp. 443–462.

[19] Buchweitz J. P., Ganey P. E., Bursian S. J., Roth R. A. (2002). Underlying
endotoxemia
augments toxic responses to chlorpromazine: is there a relationship
to drug idiosyncrasy?. J. Pharmacol Exp Ther.

[20] McGill M. R., Jaeschke H. (2019). Animal models of drug-induced
liver injury. Biochim Biophys Acta Mol. Basis
Dis.

[21] Lin C., Khetani S. R. (2016). Advances
in Engineered Liver Models for Investigating
Drug-Induced Liver Injury. Biomed. Res. Int..

[22] Greer M. L., Barber J., Eakins J., Kenna J. G. (2010). Cell based
approaches
for evaluation of drug-induced liver injury. Toxicology.

[23] Tsuchiya M., Ji C., Kosyk O., Shymonyak S., Melnyk S., Kono H., Tryndyak V., Muskhelishvili L., Pogribny I. P., Kaplowitz N., Rusyn I. (2012). Interstrain differences in liver injury and one-carbon metabolism
in alcohol-fed mice. Hepatology.

[24] Soldatow V. Y., Lecluyse E. L., Griffith L. G., Rusyn I. (2013). In vitro models for
liver toxicity testing. Toxicol Res. (Camb).

[25] Sistare F. D., Mattes W. B., LeCluyse E. L. (2016). The Promise
of New Technologies to
Reduce, Refine, or Replace Animal Use while Reducing Risks of Drug
Induced Liver Injury in Pharmaceutical Development. ILAR J..

[26] Godoy P., Hewitt N. J., Albrecht U., Andersen M. E., Ansari N., Bhattacharya S., Bode J. G., Bolleyn J., Borner C., Bottger J., Braeuning A., Budinsky R. A., Burkhardt B., Cameron N. R., Camussi G., Cho C. S., Choi Y. J., Craig Rowlands J., Dahmen U., Damm G., Dirsch O., Donato M. T., Dong J., Dooley S., Drasdo D., Eakins R., Ferreira K. S., Fonsato V., Fraczek J., Gebhardt R., Gibson A., Glanemann M., Goldring C. E., Gomez-Lechon M. J., Groothuis G. M., Gustavsson L., Guyot C., Hallifax D., Hammad S., Hayward A., Haussinger D., Hellerbrand C., Hewitt P., Hoehme S., Holzhutter H. G., Houston J. B., Hrach J., Ito K., Jaeschke H., Keitel V., Kelm J. M., Kevin Park B., Kordes C., Kullak-Ublick G. A., LeCluyse E. L., Lu P., Luebke-Wheeler J., Lutz A., Maltman D. J., Matz-Soja M., McMullen P., Merfort I., Messner S., Meyer C., Mwinyi J., Naisbitt D. J., Nussler A. K., Olinga P., Pampaloni F., Pi J., Pluta L., Przyborski S. A., Ramachandran A., Rogiers V., Rowe C., Schelcher C., Schmich K., Schwarz M., Singh B., Stelzer E. H., Stieger B., Stober R., Sugiyama Y., Tetta C., Thasler W. E., Vanhaecke T., Vinken M., Weiss T. S., Widera A., Woods C. G., Xu J. J., Yarborough K. M., Hengstler J. G. (2013). Recent advances in 2D and 3D in vitro systems using
primary hepatocytes, alternative hepatocyte sources and non-parenchymal
liver cells and their use in investigating mechanisms of hepatotoxicity,
cell signaling and ADME. Arch. Toxicol..

[27] Zhang C. J., Meyer S. R., O’Meara M. J., Huang S., Capeling M. M., Ferrer-Torres D., Childs C. J., Spence J. R., Fontana R. J., Sexton J. Z. (2023). A human
liver organoid screening platform for DILI
risk prediction. J. Hepatol.

[28] Ewart L., Apostolou A., Briggs S. A., Carman C. V., Chaff J. T., Heng A. R., Jadalannagari S., Janardhanan J., Jang K. J., Joshipura S. R., Kadam M. M., Kanellias M., Kujala V. J., Kulkarni G., Le C. Y., Lucchesi C., Manatakis D. V., Maniar K. K., Quinn M. E., Ravan J. S., Rizos A. C., Sauld J. F. K., Sliz J. D., Tien-Street W., Trinidad D. R., Velez J., Wendell M., Irrechukwu O., Mahalingaiah P. K., Ingber D. E., Scannell J. W., Levner D. (2022). Performance
assessment and economic analysis of a human Liver-Chip for predictive
toxicology. Commun. Med. (Lond.).

[29] Ribeiro A. J. S., Yang X., Patel V., Madabushi R., Strauss D. G. (2019). Liver Microphysiological Systems for Predicting and
Evaluating Drug Effects. Clin Pharmacol Ther.

[30] Gough A., Soto-Gutierrez A., Vernetti L., Ebrahimkhani M. R., Stern A. M., Taylor D. L. (2021). Human biomimetic
liver microphysiology
systems in drug development and precision medicine. Nat. Rev. Gastroenterol Hepatol.

[31] Baudy A. R., Otieno M. A., Hewitt P., Gan J., Roth A., Keller D., Sura R., Van Vleet T. R., Proctor W. R. (2020). Liver microphysiological systems development guidelines
for safety risk assessment in the pharmaceutical industry. Lab Chip.

[32] Marx U., Akabane T., Andersson T. B., Baker E., Beilmann M., Beken S., Brendler-Schwaab S., Cirit M., David R., Dehne E. M., Durieux I., Ewart L., Fitzpatrick S. C., Frey O., Fuchs F., Griffith L. G., Hamilton G. A., Hartung T., Hoeng J., Hogberg H., Hughes D. J., Ingber D. E., Iskandar A., Kanamori T., Kojima H., Kuehnl J., Leist M., Li B., Loskill P., Mendrick D. L., Neumann T., Pallocca G., Rusyn I., Smirnova L., Steger-Hartmann T., Tagle D. A., Tonevitsky A., Tsyb S., Trapecar M., Van de Water B., Van den Eijnden-van Raaij J., Vulto P., Watanabe K., Wolf A., Zhou X., Roth A. (2020). Biology-inspired microphysiological
systems to advance patient benefit and animal welfare in drug development. ALTEX.

[33] Vernetti L., Gough A., Baetz N., Blutt S., Broughman J. R., Brown J. A., Foulke-Abel J., Hasan N., In J., Kelly E., Kovbasnjuk O., Repper J., Senutovitch N., Stabb J., Yeung C., Zachos N. C., Donowitz M., Estes M., Himmelfarb J., Truskey G., Wikswo J. P., Taylor D. L. (2017). Functional Coupling
of Human Microphysiology Systems:
Intestine, Liver, Kidney Proximal Tubule, Blood-Brain Barrier and
Skeletal Muscle. Sci. Rep..

[34] Slikker W., Yuan Y., Wishart D., Wen H., Wang M., Waterton J., Wang T., Vary N., Eijnden-van-Raaij J. V. D., Tong W., Tong L., Tagle D., Sung K., Sumner S., Svendsen C. N., Silva P., Schoonjans R., Salek R. M., Rusyn I., Rodricks J. V., Ribeiro A. J., Platz S., Pirmohamed M., Pelaez C., Patri A., Mercer T., McCarthy T., Marx U., Masters S., Liu Y., Liachenko S., Krefting I., Kojima H., Kass G. E., Ishida S., Hugas M., Honma M., Hoeveler A., Hirose A., Hinton D., Halamoda-Kenzaoui B., Girard P., Fitzpatrick S., Cohen J., Beger R. D., Ball R., Bahl M. I., Anklam E. (2022). Emerging technologies
and their impact on regulatory science. Exp
Biol. Med. (Maywood).

[35] Low L. A., Mummery C., Berridge B. R., Austin C. P., Tagle D. A. (2021). Organs-on-chips:
into the next decade. Nat. Rev. Drug Discov.

[36] Ewart L., Fabre K., Chakilam A., Dragan Y., Duignan D. B., Eswaraka J., Gan J., Guzzie-Peck P., Otieno M., Jeong C. G., Keller D. A., de Morais S. M., Phillips J. A., Proctor W., Sura R., Van Vleet T., Watson D., Will Y., Tagle D., Berridge B. (2017). Navigating
tissue chips from development to dissemination: A pharmaceutical industry
perspective. Exp Biol. Med. (Maywood).

[37] Bircsak K. M., DeBiasio R., Miedel M., Alsebahi A., Reddinger R., Saleh A., Shun T., Vernetti L. A., Gough A. (2021). A 3D microfluidic
liver model for high throughput compound toxicity screening in the
OrganoPlate­(R). Toxicology.

[38] Nitsche K. S., Muller I., Malcomber S., Carmichael P. L., Bouwmeester H. (2022). Implementing organ-on-chip in a next-generation
risk
assessment of chemicals: a review. Arch. Toxicol..

[39] Jang K. J., Otieno M. A., Ronxhi J., Lim H. K., Ewart L., Kodella K. R., Petropolis D. B., Kulkarni G., Rubins J. E., Conegliano D., Nawroth J., Simic D., Lam W., Singer M., Barale E., Singh B., Sonee M., Streeter A. J., Manthey C., Jones B., Srivastava A., Andersson L. C., Williams D., Park H., Barrile R., Sliz J., Herland A., Haney S., Karalis K., Ingber D. E., Hamilton G. A. (2019). Reproducing human and cross-species
drug toxicities using a Liver-Chip. Sci. Transl.
Med..

[40] Lim A. Y., Kato Y., Sakolish C., Valdiviezo A., Han G., Bajaj P., Stanko J., Ferguson S. S., Villenave R., Hewitt P., Hardwick R. N., Rusyn I. (2023). Reproducibility and
Robustness of a Liver Microphysiological System PhysioMimix LC12 under
Varying Culture Conditions and Cell Type Combinations. Bioengineering.

[41] Cox B., Barton P., Class R., Coxhead H., Delatour C., Gillent E., Henshall J., Isin E. M., King L., Valentin J. P. (2022). Setup of
human liver-chips integrating 3D models, microwells
and a standardized microfluidic platform as proof-of-concept study
to support drug evaluation. Biomater Biosyst.

[42] Marx U., Beken S., Chen Z., Dehne E. M., Doherty A., Ewart L., Fitzpatrick S. C., Griffith L. G., Gu Z., Hartung T., Hickman J., Ingber D. E., Ishida S., Jeong J., Leist M., Levin L., Mendrick D. L., Pallocca G., Platz S., Raschke M., Smirnova L., Tagle D. A., Trapecar M., van Balkom B. W. M., van den Eijnden-van Raaij J., van der
Meer A., Roth A. (2025). Biology-inspired dynamic microphysiological system
approaches to
revolutionize basic research, healthcare and animal welfare. ALTEX.

[43] Kato Y., Lim A. Y., Sakolish C., Valdiviezo A., Moyer H. L., Hewitt P., Bajaj P., Han G., Rusyn I. (2022). Analysis of reproducibility and robustness of OrganoPlate­(R)
2-lane
96, a liver microphysiological system for studies of pharmacokinetics
and toxicological assessment of drugs. Toxicol
In Vitro.

[44] Sakolish C., Reese C. E., Luo Y. S., Valdiviezo A., Schurdak M. E., Gough A., Taylor D. L., Chiu W. A., Vernetti L. A., Rusyn I. (2021). Analysis of reproducibility and robustness
of a human microfluidic four-cell liver acinus microphysiology system
(LAMPS). Toxicology.

[45] Ewart L., Roth A. (2021). Opportunities and challenges
with microphysiological systems: a pharma
end-user perspective. Nat. Rev. Drug Discov.

[46] Rusyn I., Sakolish C., Kato Y., Stephan C., Vergara L., Hewitt P., Bhaskaran V., Davis M., Hardwick R. N., Ferguson S. S., Stanko J. P., Bajaj P., Adkins K., Sipes N. S., Hunter E. S., Baltazar M. T., Carmichael P. L., Sadh K., Becker R. A. (2022). Microphysiological
Systems Evaluation:
Experience of TEX-VAL Tissue Chip Testing Consortium. Toxicol. Sci..

[47] Rubiano A., Indapurkar A., Yokosawa R., Miedzik A., Rosenzweig B., Arefin A., Moulin C. M., Dame K., Hartman N., Volpe D. A., Matta M. K., Hughes D. J., Strauss D. G., Kostrzewski T., Ribeiro A. J. S. (2021). Characterizing the reproducibility
in using a liver microphysiological system for assaying drug toxicity,
metabolism, and accumulation. Clin Transl Sci..

[48] Rowe C., Shaeri M., Large E., Cornforth T., Robinson A., Kostrzewski T., Sison-Young R., Goldring C., Park K., Hughes D. (2018). Perfused human
hepatocyte
microtissues identify reactive metabolite-forming and mitochondria-perturbing
hepatotoxins. Toxicol In Vitro.

[49] Docci L., Milani N., Ramp T., Romeo A. A., Godoy P., Franyuti D. O., Krahenbuhl S., Gertz M., Galetin A., Parrott N., Fowler S. (2022). Exploration
and application of a
liver-on-a-chip device in combination with modelling and simulation
for quantitative drug metabolism studies. Lab
Chip.

[50] Feldhoff R.
C., Taylor J. M., Jefferson L. S. (1977). Synthesis and secretion of rat albumin
in vivo, in perfused liver, and in isolated hepatocytes. Effects of
hypophysectomy and growth hormone treatment. J. Biol. Chem..

[51] Weiner R., Hartig W., Bley T. (1991). Measurement of albumin
synthesis
in perfused rat liver using stable isotopes: [15N] hippurate as a
measure of the intracellular [15N] glycine precursor enrichment. Clin Nutr.

[52] Richmond J. E., Shoemaker W. C., Elwyn D. H. (1963). Rates of biosynthesis of plasma and
liver proteins. Am. J. Physiol..

[53] Homan K. A. (2023). Industry
Adoption of Organoids and Organs-on-Chip Technology: Toward a Paradox
of Choice. Adv. Biol. (Weinh).

[54] Bell C. C., Hendriks D. F., Moro S. M., Ellis E., Walsh J., Renblom A., Fredriksson Puigvert L., Dankers A. C., Jacobs F., Snoeys J., Sison-Young R. L., Jenkins R. E., Nordling A., Mkrtchian S., Park B. K., Kitteringham N. R., Goldring C. E., Lauschke V. M., Ingelman-Sundberg M. (2016). Characterization of primary human hepatocyte spheroids
as a model system for drug-induced liver injury, liver function and
disease. Sci. Rep..

[55] Zhong Y., Yu J. S., Wang X., Binas B., Yoo H. H. (2021). Chemical-based
primary human hepatocyte monolayer culture for the study of drug metabolism
and hepatotoxicity: Comparison with the spheroid model. FASEB J..

[56] Bell C. C., Dankers A. C. A., Lauschke V. M., Sison-Young R., Jenkins R., Rowe C., Goldring C. E., Park K., Regan S. L., Walker T., Schofield C., Baze A., Foster A. J., Williams D. P., van de
Ven A. W. M., Jacobs F., Houdt J. V., Lahteenmaki T., Snoeys J., Juhila S., Richert L., Ingelman-Sundberg M. (2018). Comparison
of Hepatic 2D Sandwich Cultures and 3D Spheroids for Long-term Toxicity
Applications: A Multicenter Study. Toxicol.
Sci..

[57] Proctor W. R., Foster A. J., Vogt J., Summers C., Middleton B., Pilling M. A., Shienson D., Kijanska M., Strobel S., Kelm J. M., Morgan P., Messner S., Williams D. (2017). Utility of
spherical human liver microtissues for prediction of clinical drug-induced
liver injury. Arch. Toxicol..

[58] Hendriks D. F., Fredriksson Puigvert L., Messner S., Mortiz W., Ingelman-Sundberg M. (2016). Hepatic 3D
spheroid models for the detection and study of compounds with cholestatic
liability. Sci. Rep..

[59] Bajaj P., Brennan R. J., Laurent S., Sauzeat S., Dufault M., Richards B., Adkins K. (2025). Transcriptomic
analysis in liver
spheroids identifies a dog-specific mechanism of hepatotoxicity for
amcenestrant. Toxicol. Sci..

[60] Borges N. C., Rezende V. M., Santana J. M., Moreira R. P., Moreira R. F., Moreno P., Borges D. C., Donato J. L., Moreno R. A. (2011). Chlorpromazine
quantification in human plasma by UPLC-electrospray ionization tandem
mass spectrometry. Application to a comparative pharmacokinetic study. J. Chromatogr B Analyt Technol. Biomed Life Sci..

[61] Gutierrez M. M., Nicolas L. B., Donazzolo Y., Dingemanse J. (2013). Relative bioavailability
of a newly developed pediatric formulation of bosentan vs. the adult
formulation. Int. J. Clin Pharmacol Ther.

[62] Bowsher R. R., Compton J. A., Kirkwood J. A., Place G. D., Jones C. D., Mabry T. E., Hyslop D. L., Hatcher B. L., DeSante K. A. (1994). Sensitive
and specific radioimmunoassay for fialuridine: initial assessment
of pharmacokinetics after single oral doses to healthy volunteers. Antimicrob. Agents Chemother..

[63] Zhang J., He K., Cai L., Chen Y. C., Yang Y., Shi Q., Woolf T. F., Ge W., Guo L., Borlak J., Tong W. (2016). Inhibition of bile
salt transport by drugs associated with liver
injury in primary hepatocytes from human, monkey, dog, rat, and mouse. Chem. Biol. Interact.

[64] Richardson F. C., Engelhardt J. A., Bowsher R. R. (1994). Fialuridine accumulates
in DNA of
dogs, monkeys, and rats following long-term oral administration. Proc. Natl. Acad. Sci. U. S. A..

[65] Wu X., Jiang D., Yang Y., Li S., Ding Q. (2023). Modeling drug-induced
liver injury and screening for anti-hepatofibrotic compounds using
human PSC-derived organoids. Cell Regen..

[66] Foster A. J., Chouhan B., Regan S. L., Rollison H., Amberntsson S., Andersson L. C., Srivastava A., Darnell M., Cairns J., Lazic S. E., Jang K. J., Petropolis D. B., Kodella K., Rubins J. E., Williams D., Hamilton G. A., Ewart L., Morgan P. (2019). Integrated in vitro
models for hepatic
safety and metabolism: evaluation of a human Liver-Chip and liver
spheroid. Arch. Toxicol..

[67] Wójcikowski J., Boksa J., Daniel W. A. (2010). Main contribution
of the cytochrome
P450 isoenzyme 1A2 (CYP1A2) to N-demethylation and 5-sulfoxidation
of the phenothiazine neuroleptic chlorpromazine in human liver–A
comparison with other phenothiazines. Biochem.
Pharmacol..

[68] MacAllister S. L., Young C., Guzdek A., Zhidkov N., O’Brien P. J. (2013). Molecular
cytotoxic mechanisms of chlorpromazine in isolated rat hepatocytes. Can. J. Physiol. Pharmacol..

[69] Trombino A. F., Near R. I., Matulka R. A., Yang S., Hafer L. J., Toselli P. A., Kim D. W., Rogers A. E., Sonenshein G. E., Sherr D. H. (2000). Expression of the aryl hydrocarbon receptor/transcription
factor (AhR) and AhR-regulated CYP1 gene transcripts in a rat model
of mammary tumorigenesis. Breast Cancer Res.
Treat.

[70] Han K. M., Ahn S. Y., Seo H., Yun J., Cha H. J., Shin J. S., Kim Y. H., Kim H., Park H. K., Lee Y. M. (2017). Bosentan and Rifampin Interactions
Modulate Influx
Transporter and Cytochrome P450 Expression and Activities in Primary
Human Hepatocytes. Biomol Ther (Seoul).

[71] Szalowska E., Stoopen G., Groot M. J., Hendriksen P. J., Peijnenburg A. A. (2013). Treatment of mouse liver slices with
cholestatic hepatotoxicants
results in down-regulation of Fxr and its target genes. BMC Med. Genomics.

[72] Anthérieu S., Bachour-El Azzi P., Dumont J., Abdel-Razzak Z., Guguen-Guillouzo C., Fromenty B., Robin M. A., Guillouzo A. (2013). Oxidative
stress plays a major role in chlorpromazine-induced cholestasis in
human HepaRG cells. Hepatology.

[73] Treiber A., Schneiter R., Hausler S., Stieger B. (2007). Bosentan is a substrate
of human OATP1B1 and OATP1B3: inhibition of hepatic uptake as the
common mechanism of its interactions with cyclosporin A, rifampicin,
and sildenafil. Drug Metab. Dispos..

[74] Wójcikowski J., Haduch A., Daniel W. A. (2012). Effect
of classic and atypical neuroleptics
on cytochrome P450 3A (CYP3A) in rat liver. Pharmacol Rep.

[75] Dingemanse J., van Giersbergen P. L. (2004). Clinical pharmacology of bosentan,
a dual endothelin
receptor antagonist. Clin Pharmacokinet.

[76] Messiha F. S. (1985). Chlorpromazine
and ethanol intoxication: an underlying mechanism. Neurobehav. Toxicol. Teratol..

[77] Hendriks D. F. G., Hurrell T., Riede J., van der Horst M., Tuovinen S., Ingelman-Sundberg M. (2019). Mechanisms of Chronic Fialuridine
Hepatotoxicity as Revealed in Primary Human Hepatocyte Spheroids. Toxicol. Sci..

[78] Jassal B., Matthews L., Viteri G., Gong C., Lorente P., Fabregat A., Sidiropoulos K., Cook J., Gillespie M., Haw R., Loney F., May B., Milacic M., Rothfels K., Sevilla C., Shamovsky V., Shorser S., Varusai T., Weiser J., Wu G., Stein L., Hermjakob H., D’Eustachio P. (2020). The reactome pathway knowledgebase. Nucleic Acids Res..

[79] Kelder T., van Iersel M. P., Hanspers K., Kutmon M., Conklin B. R., Evelo C. T., Pico A. R. (2012). WikiPathways: building research communities
on biological pathways. Nucleic Acids Res..

[80] Tsai H. D., Oware K. D., Wright F. A., Chiu W. A., Rusyn I. (2025). A workflow
for human health hazard evaluation using transcriptomic data and Key
Characteristics-based gene sets. Toxicol. Sci..

[81] Rusyn I., Arzuaga X., Cattley R. C., Corton J. C., Ferguson S. S., Godoy P., Guyton K. Z., Kaplowitz N., Khetani S. R., Roberts R. A., Roth R. A., Smith M. T. (2021). Key Characteristics
of Human Hepatotoxicants as a Basis for Identification and Characterization
of the Causes of Liver Toxicity. Hepatology.

[82] Germolec D. R., Lebrec H., Anderson S. E., Burleson G. R., Cardenas A., Corsini E., Elmore S. E., Kaplan B. L. F., Lawrence B. P., Lehmann G. M., Maier C. C., McHale C. M., Myers L. P., Pallardy M., Rooney A. A., Zeise L., Zhang L., Smith M. T. (2022). Consensus on the
Key Characteristics of Immunotoxic
Agents as a Basis for Hazard Identification. Environ. Health Perspect..

[83] Thakare R., Alamoudi J. A., Gautam N., Rodrigues A. D., Alnouti Y. (2018). Species differences in bile acids
II. Bile acid metabolism. J. Appl. Toxicol.

[84] Einarsson C., Ellis E., Abrahamsson A., Ericzon B. G., Bjorkhem I., Axelson M. (2000). Bile acid formation
in primary human hepatocytes. World J. Gastroenterol..

[85] Chiang J. Y. L., Ferrell J. M. (2018). Bile Acid Metabolism in Liver Pathobiology. Gene Expr.

[86] Pandak W. M., Ren S., Marques D., Hall E., Redford K., Mallonee D., Bohdan P., Heuman D., Gil G., Hylemon P. (2002). Transport
of cholesterol into mitochondria is rate-limiting for bile acid synthesis
via the alternative pathway in primary rat hepatocytes. J. Biol. Chem..

[87] Namdari R., Jones K., Chuang S. S., Van Cruchten S., Dincer Z., Downes N., Mikkelsen L. F., Harding J., Jackel S., Jacobsen B., Kinyamu-Akunda J., Lortie A., Mhedhbi S., Mohr S., Schmitt M. W., Prior H. (2021). Species selection for nonclinical safety assessment of drug candidates:
Examples of current industry practice. Regul.
Toxicol. Pharmacol..

[88] Regev A. (2014). Drug-induced
liver injury and drug development: industry perspective. Semin Liver Dis.

[89] Weber S., Gerbes A. L. (2022). Challenges and Future of Drug-Induced Liver Injury
Research-Laboratory Tests. Int. J. Mol. Sci..

[90] Vorrink S. U., Zhou Y., Ingelman-Sundberg M., Lauschke V. M. (2018). Prediction of Drug-Induced
Hepatotoxicity Using Long-Term Stable Primary Hepatic 3D Spheroid
Cultures in Chemically Defined Conditions. Toxicol.
Sci..

[91] Grepper S., Colombo M. V., Wen F., Fäs L., Pawlowska A., Wolf A., Filippi B. (2025). Species-Specific Liver
Microtissues: A Set of Micro-physiological Systems to Assess Translational
Hepatotoxicity in Drug Development (Abstract ID: 191855). J. Pharmacol. Exp. Ther..

[92] Vivares A., Salle-Lefort S., Arabeyre-Fabre C., Ngo R., Penarier G., Bremond M., Moliner P., Gallas J. F., Fabre G., Klieber S. (2015). Morphological behaviour and metabolic
capacity of cryopreserved
human primary hepatocytes cultivated in a perfused multiwell device. Xenobiotica.

[93] Berthiaume F., Moghe P. V., Toner M., Yarmush M. L. (1996). Effect
of extracellular
matrix topology on cell structure, function, and physiological responsiveness:
hepatocytes cultured in a sandwich configuration. FASEB J..

[94] Wang Y. J., Liu H. L., Guo H. T., Wen H. W., Liu J. (2004). Primary hepatocyte
culture in collagen gel mixture and collagen sandwich. World J. Gastroenterol.

[95] Dunn J. C., Yarmush M. L., Koebe H. G., Tompkins R. G. (1989). Hepatocyte function
and extracellular matrix geometry: long-term culture in a sandwich
configuration. FASEB J..

[96] de
Bruijn V. M. P., Wang Z., Bakker W., Zheng W., Spee B., Bouwmeester H. (2022). Hepatic bile acid synthesis and secretion:
Comparison of in vitro methods. Toxicol. Lett..

[103] Chen S. (2018). fastp: an ultra-fast all-in-one FASTQ
preprocessor. Bioinformatics.

[97] Dobin A., Davis C. A., Schlesinger F., Drenkow J., Zaleski C., Jha S., Batut P., Chaisson M., Gingeras T. R. (2013). STAR: ultrafast
universal RNA-seq aligner. Bioinformatics.

[98] Danecek P., Bonfield J. K., Liddle J., Marshall J., Ohan V., Pollard M. O., Whitwham A., Keane T., McCarthy S. A., Davies R. M., Li H. (2021). Twelve years
of SAMtools and BCFtools. Gigascience.

[99] Anders S., Pyl P. T., Huber W. (2015). HTSeq–a
Python framework to
work with high-throughput sequencing data. Bioinformatics.

[100] Love M. I., Huber W., Anders S. (2014). Moderated
estimation
of fold change and dispersion for RNA-seq data with DESeq2. Genome Biol..

[101] Yu G., He Q. Y. (2016). ReactomePA: an R/Bioconductor
package for reactome
pathway analysis and visualization. Mol. Biosyst.

[102] Yu G., Wang L. G., Han Y., He Q. Y. (2012). clusterProfiler:
an R package for comparing biological themes among gene clusters. OMICS.

