# Supplementary material for: Comparative Analysis of Species-Specific Hepatocyte Function and Drug Effects in a Liver Microphysiological System PhysioMimix LC12 and 96-Well Plates
Source: ACS Pharmacol Transl Sci. 2025 Oct 3;8(11):4138–58. doi: 10.1021/acsptsci.5c00554 (PMC12624441; doi:10.1021/acsptsci.5c00554)

Supplemental Materials for

Comparative Analysis of Species-Specific Hepatocyte Function and Drug Effects in a Liver Microphysiological System PhysioMimix® LC12 and 96-Well Plates

Chander K. Negi[1], Courtney Sakolish[1], Han-Hsuan D. Tsai[1], Katharina Nitsche[1,2], Han Gang[3], Piyush Bajaj[4], Stephen S. Ferguson[5], Jason P. Stanko[5], Philip Hewitt[6], David A. Kukla[7], Sarah M. Lloyd[7], Remi Villenave[8] and Ivan Rusyn[1,*]

[1]Department of Veterinary Physiology and Pharmacology, Texas A&M University, College Station, TX, USA
[2]Division of Toxicology, Wageningen University and Research, Wageningen, Gelderland 6708 WE, the Netherlands
[3]Department of Epidemiology and Biostatistics, Texas A&M University, College Station, TX 77843, USA
[4]Global Investigative Toxicology, Preclinical Safety, Sanofi, Cambridge, MA 02141, USA
[5]Division of Translational Toxicology, National Institute of Environmental Health Sciences, Research Triangle Park, NC 27709, USA
[6]Chemical and Preclinical Safety, Merck KGaA, Darmstadt, 64293, Germany
[7]Development Sciences, AbbVie Inc., North Chicago, IL 60064, USA
[8]Roche Pharma Research and Early Development, Roche Innovation Center Basel, F. Hoffmann-La Roche Ltd, 4070 Basel, Switzerland

***Corresponding author:** Ivan Rusyn, MD, PhD, Department of Veterinary Physiology and Pharmacology, Texas A&M University, College Station, TX 77843-4466. Phone +1-979-458-9866. Email: irusyn@tamu.edu

Table of contents

Table S1. Sample size estimates for observing time-related differences in albumin and urea levels in experiments with human (H), monkey (M), rat (R), and dog (D) hepatocytes cultured in 96-well plates or PhysioMimix LC12 MPS…………………………………………………...…..S4

Table S2. The number of samples needed to observe a significant difference (with 80% power and significance level 0.05 based on the paired Cohen's D test) between time points for LDH release into the media…………………………………………………………………………….S5

Table S3. Sample size estimates for observing time-related differences in compounds-induced toxicity in experiments with PhysioMimix LC12 seeded with primary hepatocytes from human, monkey, rat, and dog……………………………………………………………………..S6

Table S4. Normalized gene count data (Supplemental table for figure 4A)………………………S7

Table S5. Normalized gene count data (Supplemental table for figure 4B)……….………………S7

Table S6. Full list of overlapping pathways (Supplemental table for Figure 5B, C and D)………S7

Table S7. Log2-fold change values of ADME genes (Supplemental table for Figure 7E)………..S7

Table S8. Full list of enriched pathways after drug treatment (Supplemental table for Figure 8)…S7

Table S9. List of studies used in the Figures with links to the raw data in EveAnalytics Database..S8

Figure S1. Comparison of functional differences (Albumin and Urea secretion) of hepatocytes in 2D static culture (96-well plates) and PhysioMimix LC12 MPS and on day 4 of culture………S9

Figure S2. Intra-experimental variability (expressed as CV) of functional parameters albumin and urea when using the PhysioMimix LC12 and 96 well plates…………………………………….S10

Figure S3. Time course of LDH release as a stress marker in 2D static culture (96 well plates) and PhysioMimix LC12 MPS….……………………………………………………………….....S11

Figure S4. Time-course analysis of liver toxicity biomarkers (AST) in 2D static culture (96 well plates) PhysioMimix LC12 MPS……………………………………………………………S12

Figure S5. Time-course analysis of liver toxicity biomarkers (ALT) in 2D static culture (96 well plates) and PhysioMimix LC12 MPS………………………………………………………...S13

Figure S6. Assessment of known DILI-causing drugs on hepatocytes function and injury markers in 2D cultures……………………………………………………………………………...S14

Figure S7. Assessment of known DILI-causing drugs on hepatocytes function and injury markers in 2D cultures on 2nd day of exposure……………………………………………………………..S15

Figure S8. Assessment of known DILI-causing drugs on hepatocytes function and injury markers in PhysioMimix LC12……………………...……………………………………………S16

Figure S9. Assessment of known DILI-causing drugs on hepatocytes function and injury markers in PhysioMimix LC12 treatment with 10 µM of CPZ, BOS and FIAU for 4 days………………S17

Figure S10. Assessment of known DILI-causing drugs on hepatocytes function and injury markers in PhysioMimix LC12 treatment with 30 µM of CPZ, BOS and FIAU for 10 days……………..S18

Figure S11. Time-course analysis of bile acid secreted by untreated cells at 4, 8 and 14 days of the experiment using the PhysioMimix™ LC12 system..…………………………………….….S19

Table S1. Sample size estimates for observing time-related differences in albumin and urea levels in experiments with human (H), monkey (M), rat (R), and dog (D) hepatocytes cultured in 96-well plates or PhysioMimix® LC12 MPS. Shown are the number of replicates (wells of devices) needed for detecting significant ($p < 0.05$ with 80% power) differences between timepoints.

	**Albumin**				**Urea**			
	**H**	**M**	**R**	**D**	**H**	**M**	**R**	**D**
96-Well Plates								
Day 4 vs Day 6	14	7	>100	8	9	8	>100	7
Day 4 vs Day 8	5	5	53	7	5	4	10	6
PhysioMimix® LC12 MPS								
Day 4 vs Day 6	8	7	>100	6	5	4	4	4
Day 4 vs Day 8	8	5	>100	6	5	3	4	3
Day 4 vs Day 10	7	6	>100	5	5	3	3	3
Day 4 vs Day 12	7	5	18	5	4	3	3	3
Day 4 vs Day 14	7	5	11	5	4	3	3	3

Table S2. The number of samples needed to observe a significant difference (with 80% power and significance level 0.05 based on the paired Cohen's D test between time points for LDH release into the media. Data is shown separately for human (H), monkey (M), rat (R), and dog (D) hepatocytes cultured in 96-well plates or PhysioMimix® LC12 MPS.

	LDH			
	H	M	R	D
96-Well Plates				
Day 6 vs Day 8	14	21	89	15
PhysioMimix® LC12 MPS				
Day 6 vs Day 8	4	42	5	6
Day 6 vs Day 10	4	12	7	5
Day 6 vs Day 12	4	13	4	5
Day 6 vs Day 14	4	10	4	4

Table S3. Sample size estimates for observing time-related differences in compounds-induced toxicity in experiments with PhysioMimix® LC12 seeded with primary hepatocytes from human, monkey, rat, and dog. Shown are the number of replicate chips needed for detecting significant ($p < 0.05$ with 80% power) differences between timepoints or in a trend test for LDH and albumin endpoints. Abbreviations: CPZ, chlorpromazine; BOS, bosentan; FIAU, fialuridine; H, human; M, monkey; R, rat; D, dog. Asterisks indicate instances where no time-dependent trend was observed.

	CPZ				BOS				FIAU			
	Albumin											
	H	M	R	D	H	M	R*	D	H	M*	R	D
Day 6	>100	51	81	18	>100	>100	58	34	23	51	>100	>100
Day 8	8	12	24	14	6	32	71	>100	8	75	47	38
Day 10	>100	8	12	5	3	>100	>100	17	5	>100	73	9
Day 12	4	3	7	4	2	24	>100	7	3	>100	52	7
Day 14	2	3	5	5	2	5	92	6	2	88	25	7
	LDH											
	H	M*	R	D*	H	M*	R*	D*	H*	M*	R	D*
Day 6	>100	51	23	>100	15	9	10	>100	13	26	12	44
Day 8	>100	13	13	3	8	66	40	14	31	61	>100	57
Day 10	6	>100	>100	>100	2	>100	>100	>100	34	>100	>100	>100
Day 12	5	7	>100	10	3	5	>100	47	33	4	>100	20
Day 14	3	>100	>100	5	4	33	>100	9	19	>100	>100	27

Table S4. Normalized gene count data (Supplemental table for figure 4A) - (spreadsheet)

Table S5. Normalized gene count data (Supplemental table for figure 4B) - (spreadsheet)

Table S6. Full list of overlapping pathways (Supplemental table for figure Figure SB, C and D) - (spreadsheet)

Table S7. Log_2 fold change values of ADME genes (Supplemental table for Figure 7E) - (spreadsheet)

Table S8. Full list of enriched pathways after drug treatment (Supplemental table for Figure 8) - (spreadsheet)

Table S9: List of studies used in the Figures with links to the raw data in EveAnalytics Database.

Figure	Study Name in the MPS-Database	URLs
3	Cross-species comparison, 96 well plates (Experiment 1)<br>Cross-species comparison, PhysioMimix® LC12 (Experiment 1)<br>Cross-species comparison, PhysioMimix® LC12 (Experiment 2)<br>Cross-species comparison, 96 well plates (Experiment 2)<br>Cross-species comparison, 96 well plates (Experiment 3)<br>Cross-species comparison, PhysioMimix® LC12 (Experiment 3)<br>Cross-species comparison, 96 well plates (Experiment 4)<br>Cross-species comparison, PhysioMimix® LC12 (Experiment 4)<br>Cross-species comparison, PhysioMimix® LC12 and 96 well plates (Experiment 5)	https://eve.eveanalytics.com/assays/assaystudy/1405/<br>https://eve.eveanalytics.com/assays/assaystudy/1406/<br>https://eve.eveanalytics.com/assays/assaystudy/1408/<br>https://eve.eveanalytics.com/assays/assaystudy/1404/<br>https://eve.eveanalytics.com/assays/assaystudy/1402/<br>https://eve.eveanalytics.com/assays/assaystudy/1393/<br>https://eve.eveanalytics.com/assays/assaystudy/1401/<br>https://eve.eveanalytics.com/assays/assaystudy/1392/<br>https://eve.eveanalytics.com/assays/assaystudy/1407/
5	Cross-species comparison, 96 well plates (Experiment 1)<br>Cross-species comparison, 96 well plates (Experiment 2)<br>Cross-species comparison, 96 well plates (Experiment 3)<br>Cross-species comparison, 96 well plates (Experiment 4)	https://eve.eveanalytics.com/assays/assaystudy/1405/<br>https://eve.eveanalytics.com/assays/assaystudy/1404/<br>https://eve.eveanalytics.com/assays/assaystudy/1402/<br>https://eve.eveanalytics.com/assays/assaystudy/1401/
6	Cross-species comparison, PhysioMimix® LC12 (Experiment 3)<br>Cross-species comparison, PhysioMimix® LC12 (Experiment 4)	https://eve.eveanalytics.com/assays/assaystudy/1393/<br>https://eve.eveanalytics.com/assays/assaystudy/1392/
9	Bile acids	https://eve.eveanalytics.com/assays/assaystudy/1484/

Figure S1. Comparison of functional differences (Albumin and Urea secretion) of hepatocytes in 2D static culture (96-well plates) and MPS PhysioMimix LC12 and on day 4 of culture. Data are presented as box plots representing the 10th to 90th percentiles, $p < 0.001$ (***), n= 5 to 12 from 5 independent experiments.

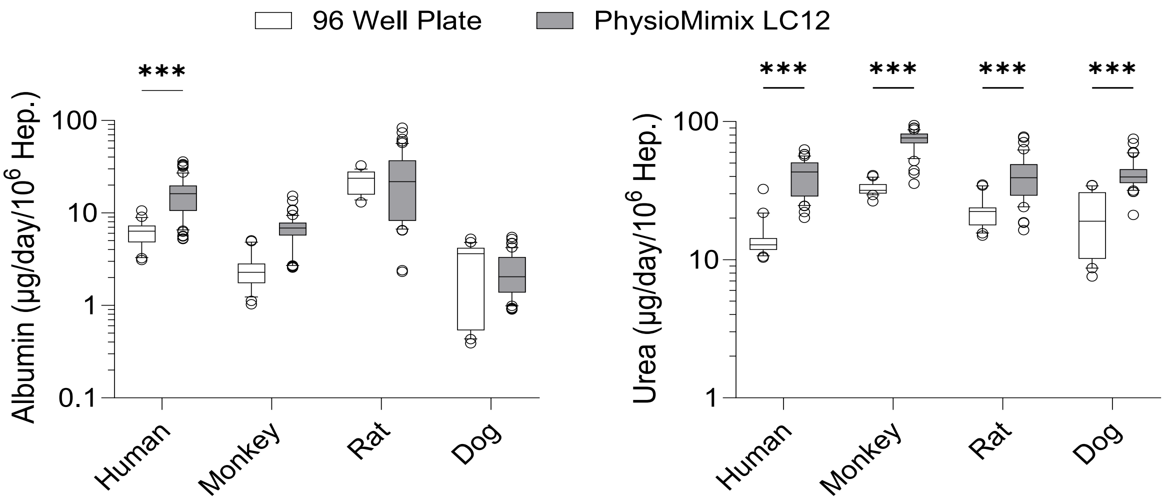

Figure S2. Intra-experimental variability (expressed as CV) of functional parameters albumin and urea when using the PhysioMimix LC12 and 96 well plates. Colors indicate data from separate experiments (see Figure 1 and Methods for details). The line is fit to all data.

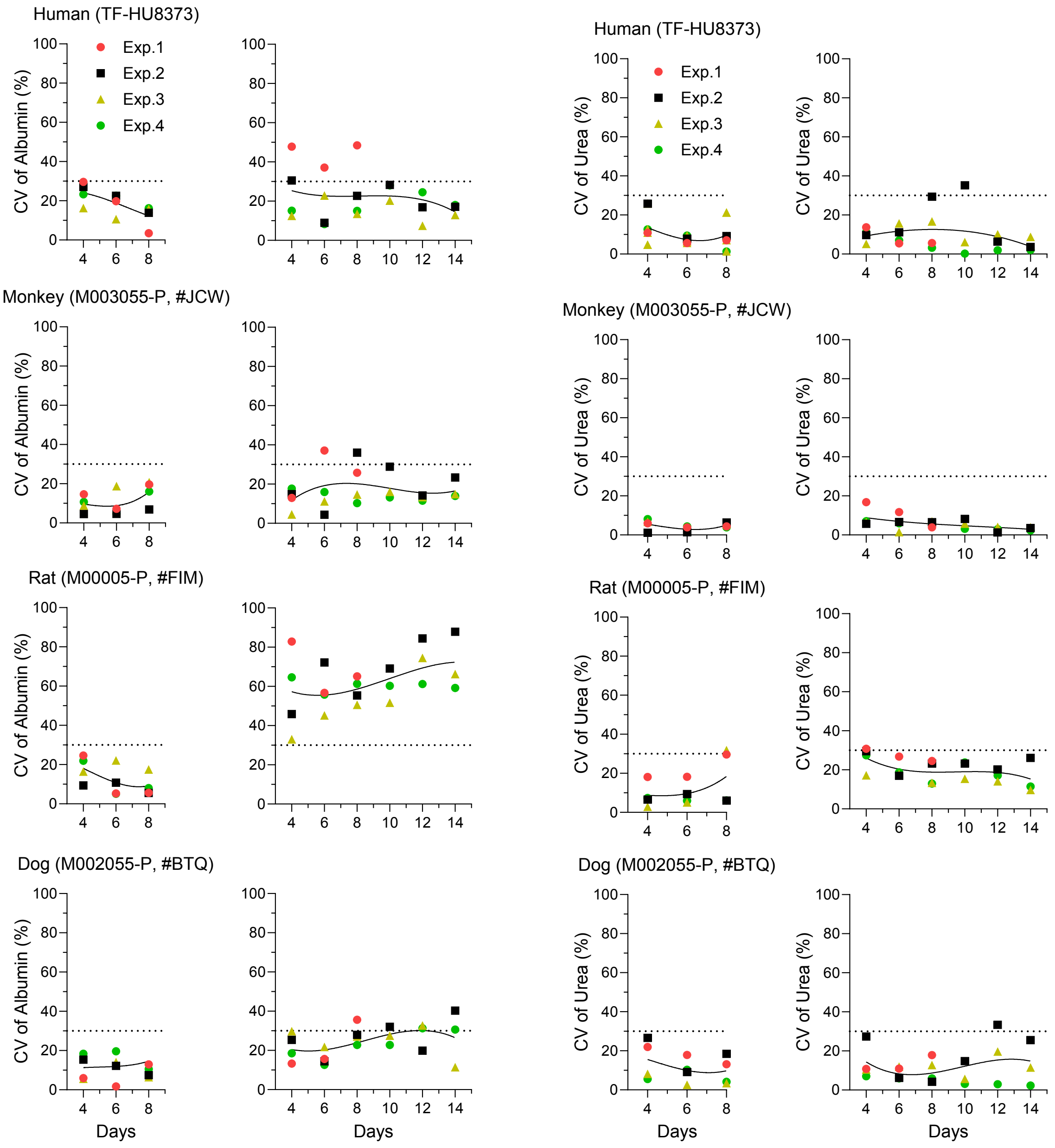

Figure S3. Time course of LDH release as a stress marker in 2D static culture (96 well plates) and MPS PhysioMimix LC12. The box plot shows the median, interquartile range (IQR), and 10th–90th percentiles. Intra-experimental variability (expressed as CV) of stress marker LDH when using the 2D static culture (96 well plates) and PhysioMimix LC12.

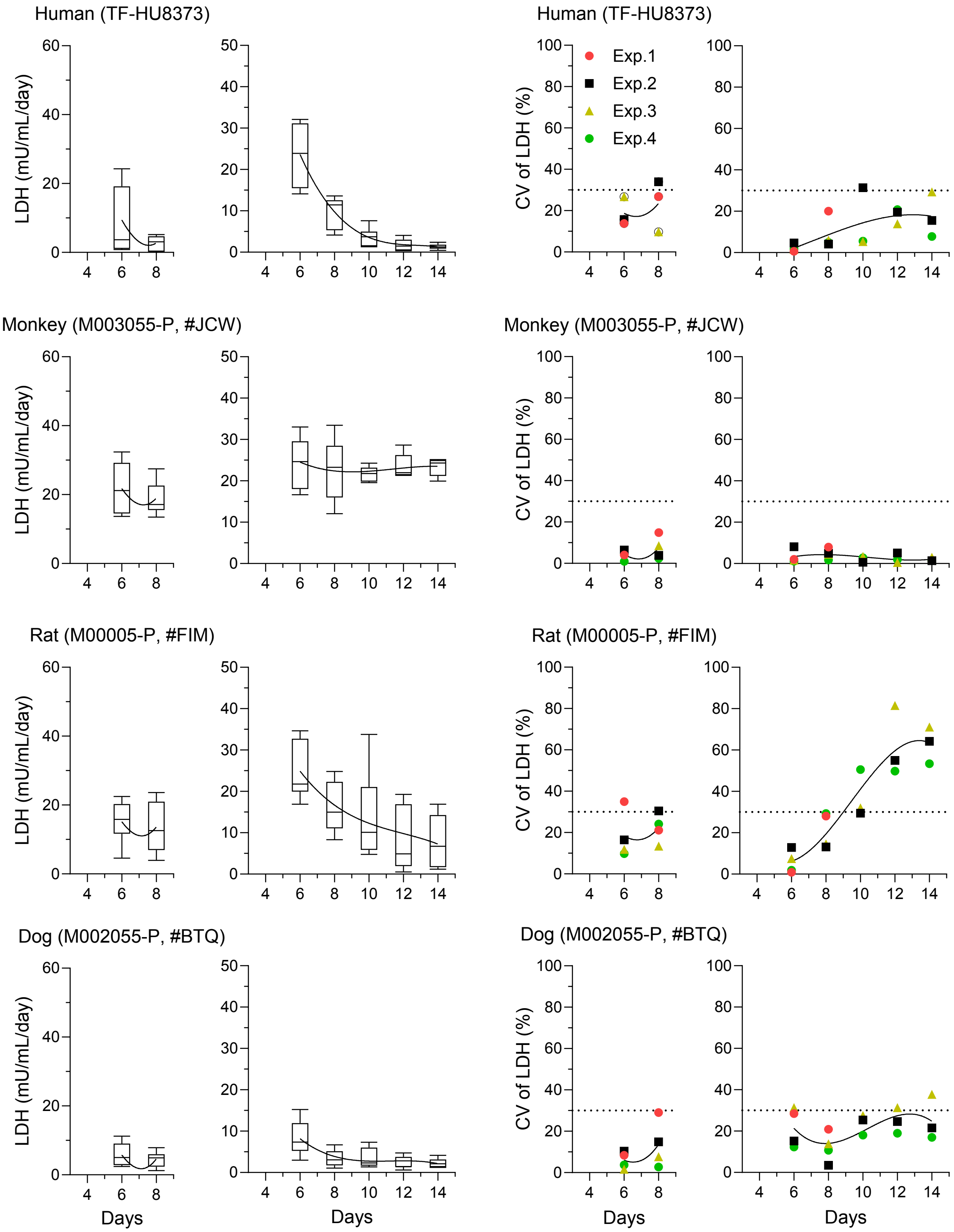

Figure S4. Time-course analysis of liver toxicity biomarkers (AST) in 2D static culture (96 well plates) MPS PhysioMimix LC12 system. The box plot represents the median, interquartile range (IQR), and 10th–90th percentiles, illustrating the distribution and variability of AST levels across different experimental conditions. Intra-experimental variability, expressed as the coefficient of variation (CV) in both the 2D static culture (96 well plates) and the PhysioMimix LC12 system.

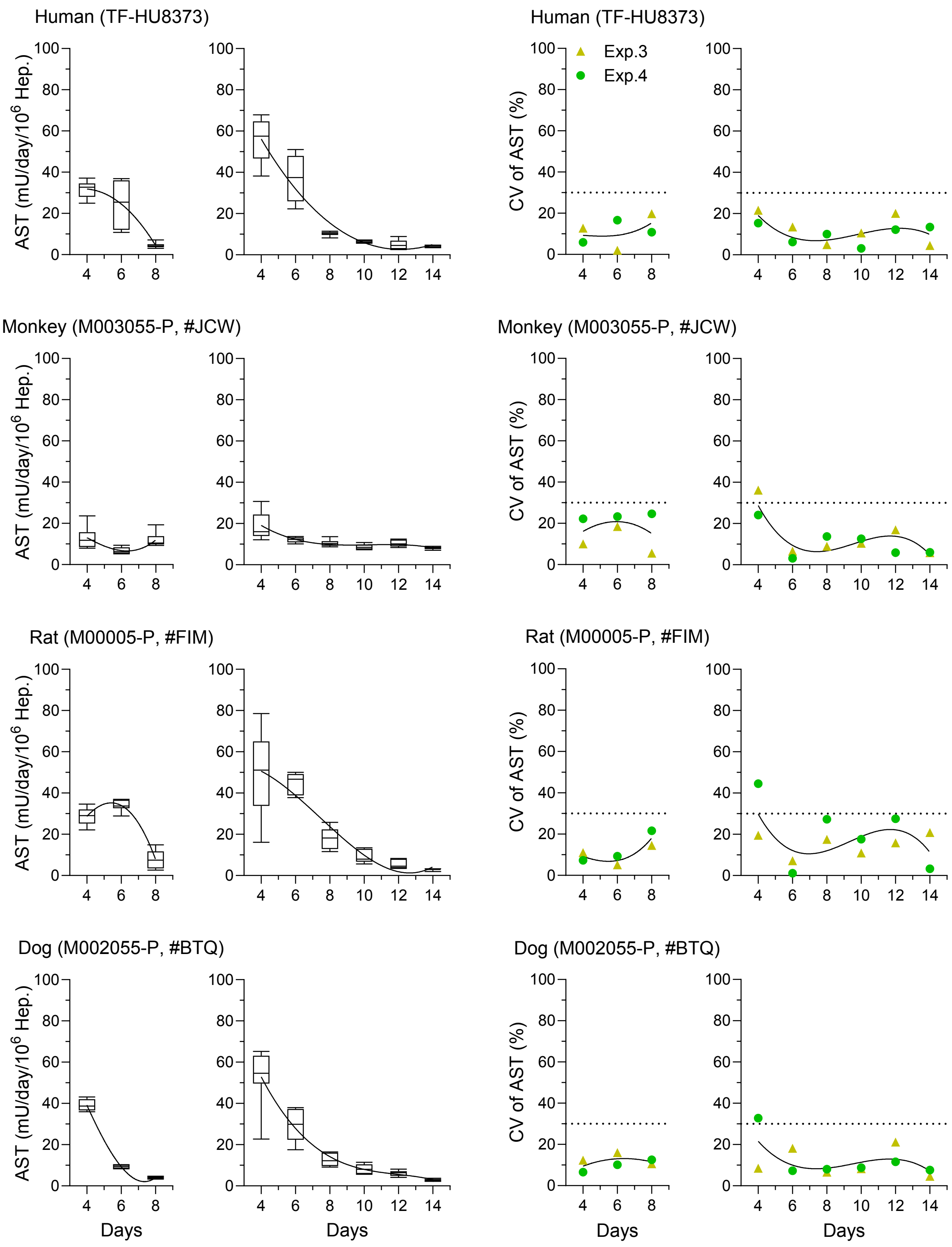

Figure S5. Time-course analysis of liver toxicity biomarkers (ALT) in 2D static culture (96 well plates) and MPS PhysioMimix LC12. The box plot represents the median, interquartile range

(IQR), and 10th–90th percentiles. Intra-experimental variability, expressed as the coefficient of variation (CV), was in both the 2D static culture and the PhysioMimix LC12 system.

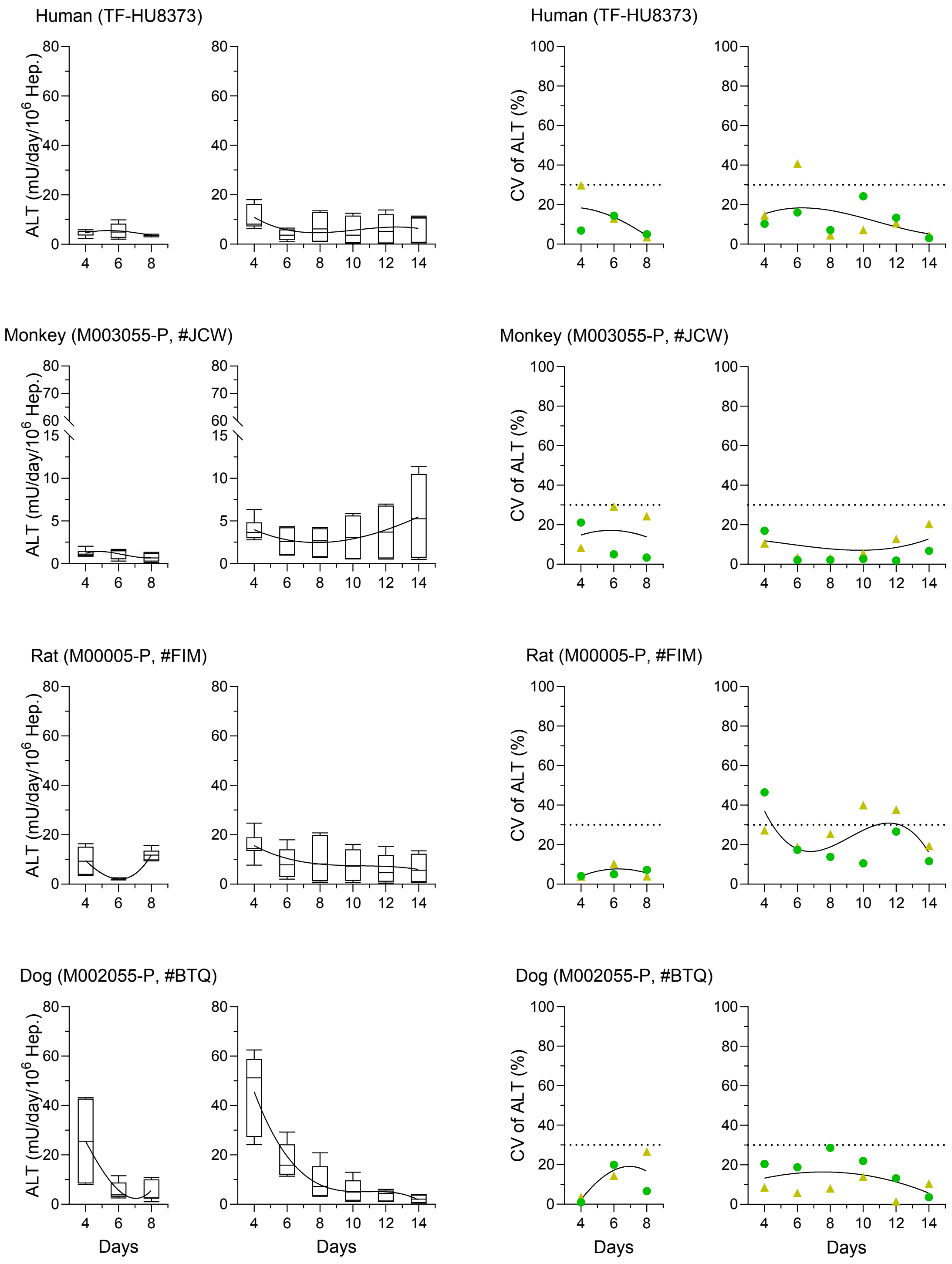

Figure S6. Assessment of known DILI-causing drugs on hepatocytes function and injury markers in 2D cultures. Shown are box-and-whiskers plots indicating interquartile range and min-max values with individual well data shown as dots. Data for hepatocytes from humans (blue), monkeys (red), rats (green) and dogs (purple) are shown for CPZ (left), BOS (middle) and FIAU (right). Concentration-response (in μM) is shown, and data are from the last day of exposure. See the heatmap showing these data in Figure 5 of the manuscript.

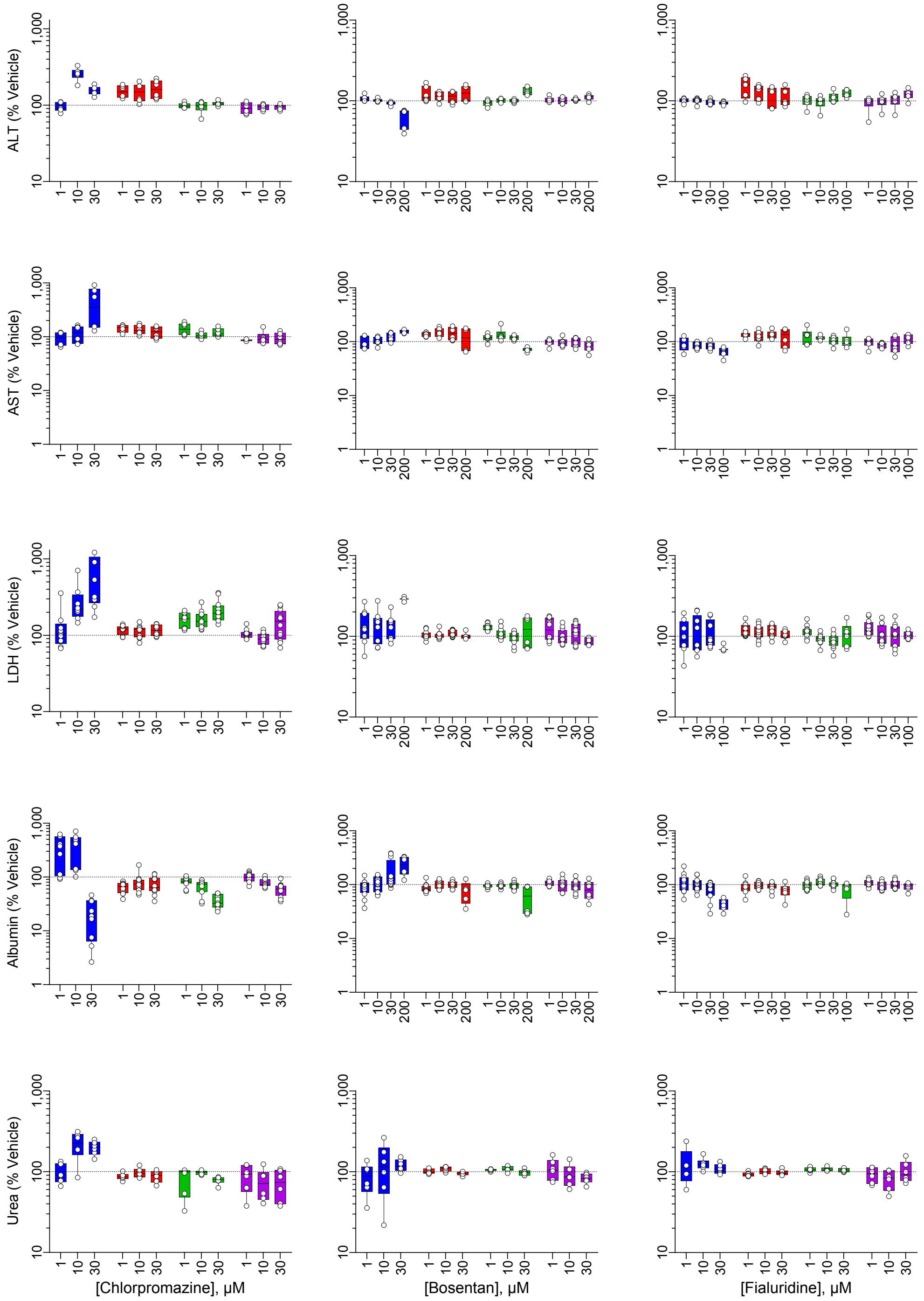
ALT (% Vehicle)
AST (% Vehicle)
LDH (% Vehicle)
Albumin (% Vehicle)
Urea (% Vehicle)
[Chlorpromazine], µM
[Bosentan], µM
[Fialuridine], µM

Figure S7. Assessment of known DILI-causing drugs on hepatocytes function and injury markers in 2D cultures on 2nd day of exposure. Shown are box-and-whiskers plots indicating interquartile range and min-max values with individual MPS data shown as dots. Data for hepatocytes from humans (blue), monkeys (red), rats (green) and dogs (purple) are shown for CPZ (left), BOS (middle) and FIAU (right).

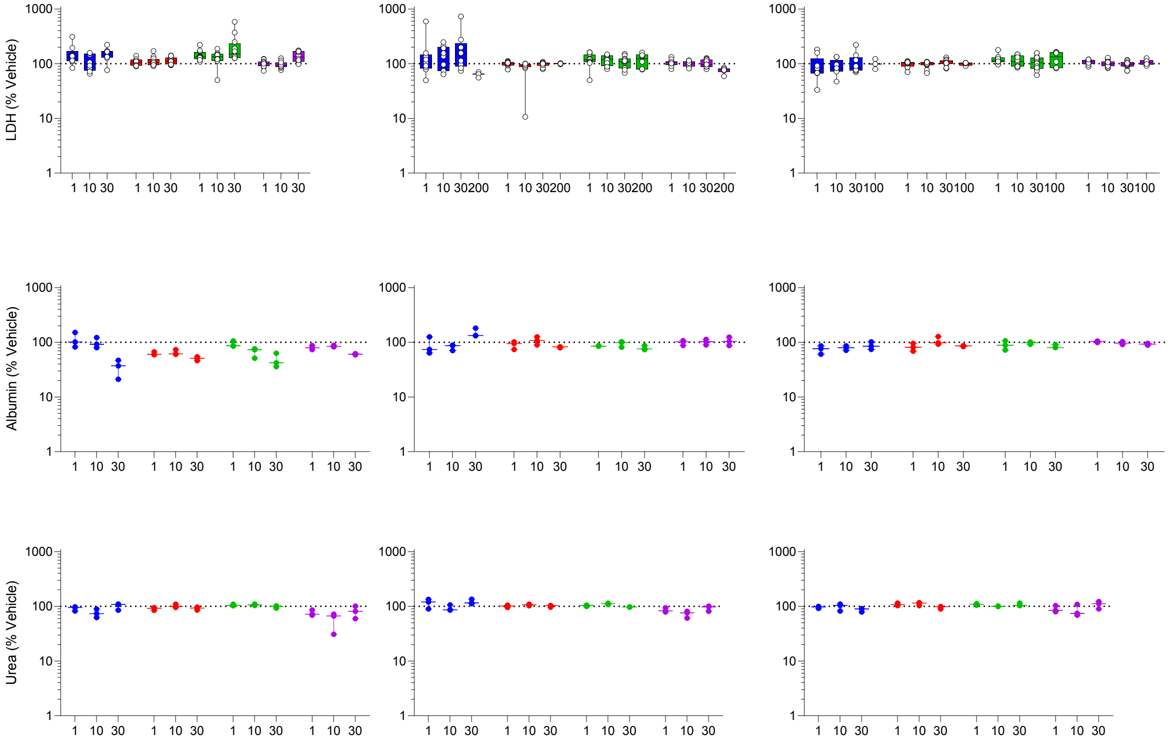

Figure S8. Assessment of known DILI-causing drugs on hepatocytes function and injury markers in PhysioMimix LC12. Shown are box-and-whiskers plots indicating interquartile range and min-max values with individual MPS data shown as dots. Data for hepatocytes from humans (blue), monkeys (red), rats (green) and dogs (purple) are shown for CPZ (left), BOS (middle) and FIAU (right). Time-course (days of treatment) is shown for repeat-dose treatments. See the heatmap showing these data in Figure 6 of the manuscript.

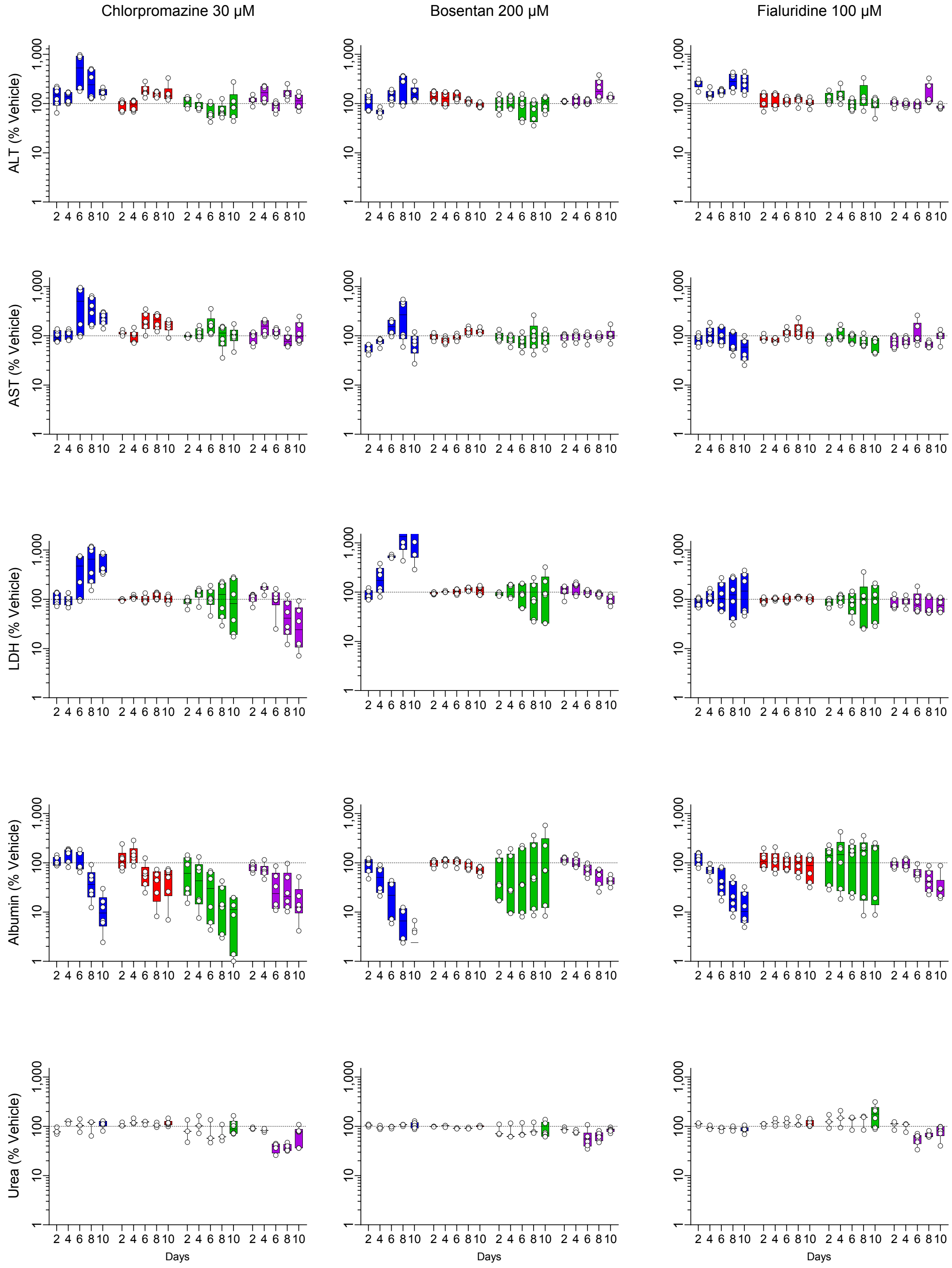

Figure S9. Assessment of known DILI-causing drugs on hepatocytes function and injury markers in PhysioMimix LC12 treatment with 10 μM of CPZ, BOS and FIU for 4 days. Shown are box-and-whiskers plots indicating interquartile range and min-max values with individual MPS data shown as dots. Data for hepatocytes from humans (blue), monkeys (red), rats (green) and dogs (purple) are shown for CPZ (left), BOS (middle) and FIAU (right). Time-course (days of treatment) is shown for repeat-dose treatments.

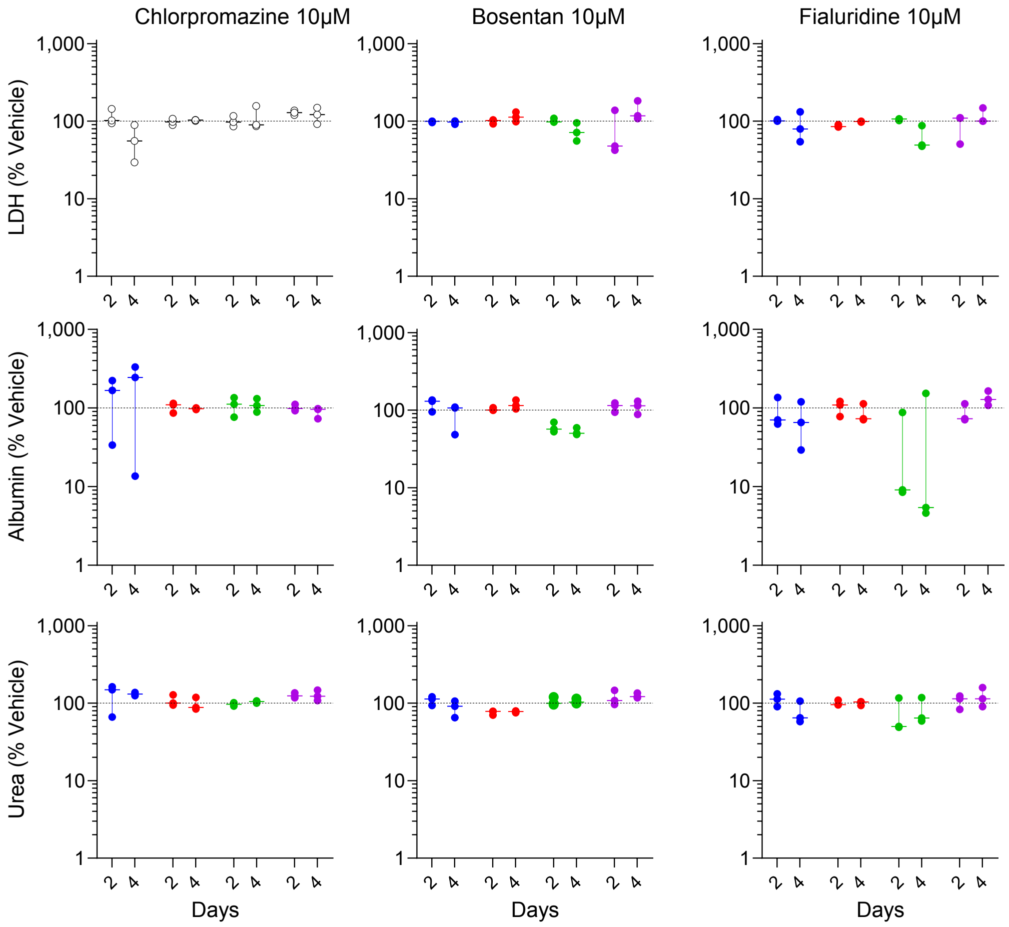

Figure S10. Assessment of known DILI-causing drugs on hepatocytes function and injury markers in PhysioMimix LC12 treatment with 30 µM of CPZ, BOS and FIAU for 10 days. Shown are box-and-whiskers plots indicating interquartile range and min-max values with individual MPS data shown as dots. Data for hepatocytes from humans (blue), monkeys (red), rats (green) and dogs (purple) are shown for CPZ (left), BOS (middle) and FIAU (right). Time-course (days of treatment) is shown for repeat-dose treatments.

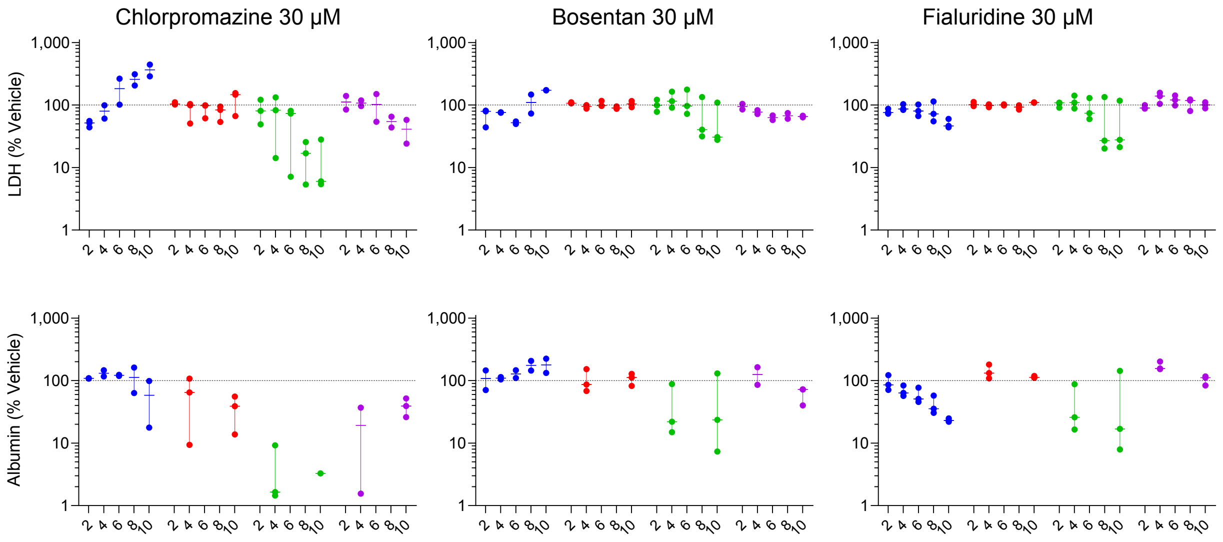

Figure S11. Time-course analysis of bile acid secreted by untreated cells at 4, 8 and 14 days of the experiment using the PhysioMimix™ LC12 system. Data is presented as box-and-whiskers plots from 3-6 replicates samples indicating interquartile range and min-max values with individual MPS data shown as dots. Abbreviations: CDCA, Chenodeoxycholic acid; GCDCA, Glycochenodeoxycholic acid; TCDCA, Taurochenodeoxycholic acid; CA, Cholic acid; GCA, Glycocholic acid; TCA, taurocholic acid.

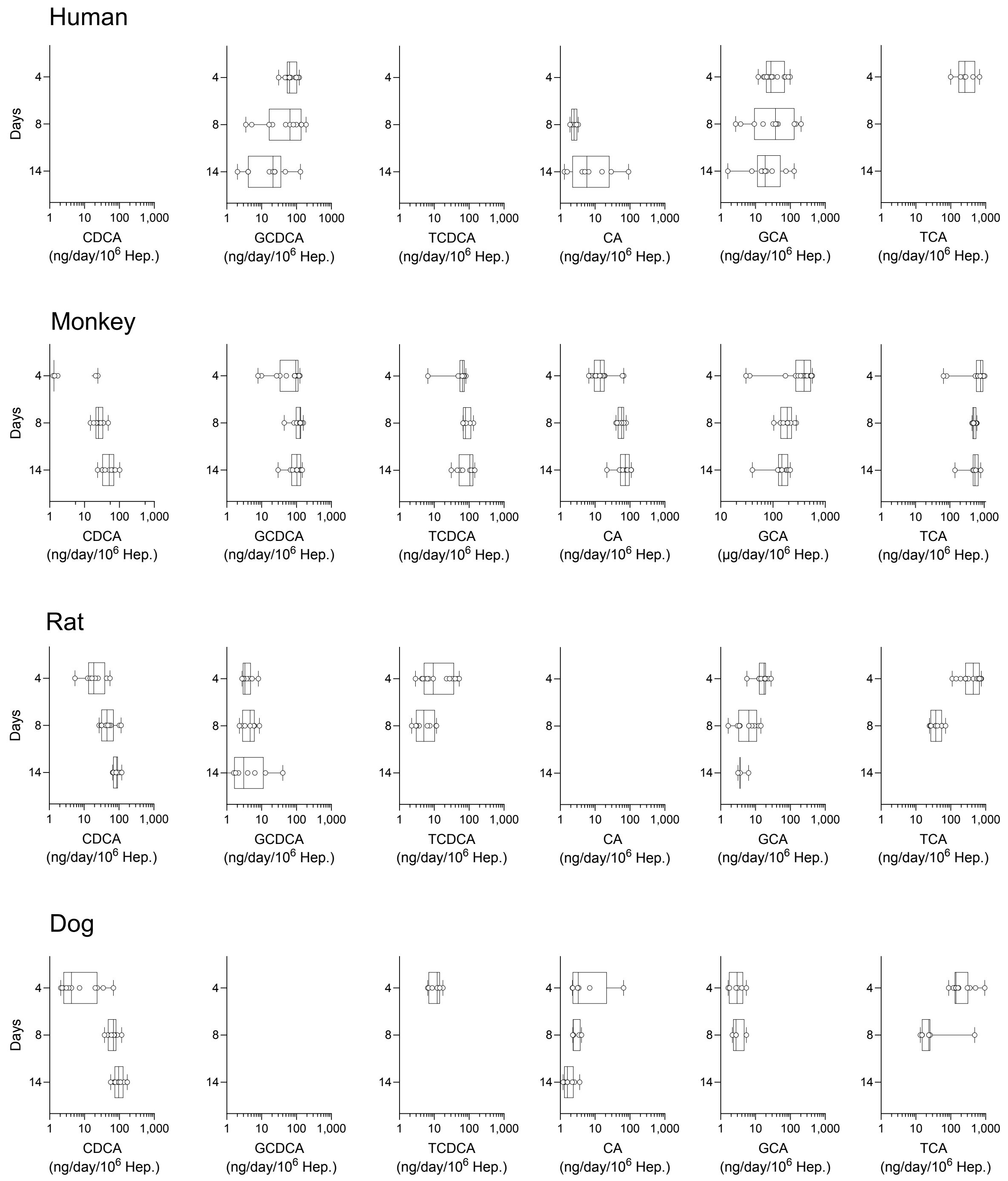

Supplement: Supplementary file 1 [file pt5c00554_si_001.pdf]
